# *In Vitro* Pro-apoptotic and Anti-migratory Effects of *Ficus deltoidea* L. Plant Extracts on the Human Prostate Cancer Cell Lines PC3

**DOI:** 10.3389/fphar.2017.00895

**Published:** 2017-12-12

**Authors:** Mohd M. M. Hanafi, Adlin Afzan, Harisun Yaakob, Ramlan Aziz, Mohamad R. Sarmidi, Jean-Luc Wolfender, Jose M. Prieto

**Affiliations:** ^1^Department of Pharmaceutical and Biological Chemistry, UCL School of Pharmacy, London, United Kingdom; ^2^Phytochemistry and Bioactive Natural Product, University of Geneva and University of Lausanne, Geneva, Switzerland; ^3^Herbal Medicine Research Centre, Institute for Medical Research (IMR), Ministry of Health Malaysia, Kuala Lumpur, Malaysia; ^4^Institute of Bioproduct Development (IBD), Universiti Teknologi Malaysia, Johor Bahru, Malaysia; ^5^Faculty of Chemical and Energy Engineering, Universiti Teknologi Malaysia, Johor Bahru, Malaysia

**Keywords:** apoptosis, migration, invasion, PC3, LNCaP, prostate cancer, *Ficus deltoidea*

## Abstract

This study aims to evaluate the *in vitro* cytotoxic and anti-migratory effects of *Ficus deltoidea* L. on prostate cancer cells, identify the active compound/s and characterize their mechanism of actions. Two farmed varieties were studied, var. *angustifolia* (FD1) and var. *deltoidea* (FD2). Their crude methanolic extracts were partitioned into *n*-hexane (FD1h, FD2h) chloroform (FD1c, FD2c) and aqueous extracts (FD1a, FD2a). Antiproliferative fractions (IC50 < 30 μg/mL, SRB staining of PC3 cells) were further fractionated. Active compound/s were dereplicated using spectroscopic methods. *In vitro* mechanistic studies on PC3 and/or LNCaP cells included: annexin V-FITC staining, MMP depolarization measurements, activity of caspases 3 and 7, nuclear DNA fragmentation and cell cycle analysis, modulation of Bax, Bcl-2, Smac/Diablo, and Alox-5 mRNA gene expression by RT-PCR. Effects of cytotoxic fractions on 2D migration and 3D invasion were tested by exclusion assays and modified Boyden chamber, respectively. Their mechanisms of action on these tests were further studied by measuring the expression VEGF-A, CXCR4, and CXCL12 in PC3 cells by RT-PCR. FD1c and FD2c extracts induced cell death (*P* < 0.05) via apoptosis as evidenced by nuclear DNA fragmentation. This was accompanied by an increase in MMP depolarization (*P* < 0.05), activation of caspases 3 and 7 (*P* < 0.05) in both PC3 and LNCaP cell lines. All active plant extracts up-regulated Bax and Smac/DIABLO, down-regulated Bcl-2 (*P* < 0.05). Both FD1c and FD2c were not cytotoxic against normal human fibroblast cells (HDFa) at the tested concentrations. Both plant extracts inhibited both migration and invasion of PC3 cells (*P* < 0.05). These effects were accompanied by down-regulation of both VEGF-A and CXCL-12 gene expressions (*P* < 0.001). LC–MS dereplication using taxonomy filters and molecular networking databases identified isovitexin in FD1c; and oleanolic acid, moretenol, betulin, lupenone, and lupeol in FD2c. In conclusion, FD1c and FD2c were able to overcome three main hallmarks of cancer in PC3 cells: (1) apoptosis by activating of the intrinsic pathway, (2) inhibition of both migration and invasion by modulating the CXCL12-CXCR4 axis, and (3) inhibiting angiogenesis by modulating VEGF-A expression. Moreover, isovitexin is here reported for the first time as an antiproliferative principle (IC50 = 43 μg/mL, SRB staining of PC3 cells).

## Introduction

Prostate cancer is considered as one of the most common types of cancer in the world, which affect mostly in the western societies. It is estimated that, 1.1 million men worldwide were diagnosed with prostate cancer in 2012, which account for almost 15% of the cancers diagnosed in men. 70% of these cases occurred in more developed nations and this could be due to the practice of prostate specific antigen (PSA) testing and subsequent biopsy that has become widespread in this part of the world. 307,000 estimated deaths were reported in 2012 which make prostate cancer as the fifth leading cause of death from cancer in men (6.6% of the total men deaths) (Ferlay et al., 2013). In the United Kingdom, prostate cancer contributed to the 46,690 (13%) new cases and 11,287 deaths annually. Almost half, 54%, of the prostate cancer cases in the United Kingdom are diagnosed in males aged 70 and over every year (Cancer Research UK, 2016). According to the statistics provided by the National Cancer Registry, Ministry of Health Malaysia (2011) of Malaysia, prostate cancer ranked ninth overall and is the fourth most frequent cancer (7.3% of all cancers) diagnosed in men. Although the incidence of prostate cancer is more prevalent in the western countries, the number of prostate cancer cases has also grown rapidly in Asian countries such as Japan, and Hong Kong (Wynder et al., 1991; Liu et al., 1993). Initially, this phenomenon was linked to the influx of Western food restaurants and franchises in most of the Asian continent that have led to the change of the normal dietary pattern of Asians. However, numerous studies made clear that even though diet could be one of the risk factors for prostate cancer, it only has a week association with the disease. Direct risk factors such as age, ethnicity, family history, and genetic conditions are reported to play major role in the onset of prostate cancer.

Approximately 90% of androgen-dependent prostate cancer is detected either locally or at regional stages, thus explaining the high cure rate, as nearly 100% of men diagnosed and treated at this stage will be disease free after 5 years. However, many men die as therapy eventually fails when the disease progresses to androgen-independent stage (Feldman and Feldman, 2001). This stage of prostate cancer is lethal and has the ability to progress and metastasize. At present, there is no effective therapy that can be used for men diagnosed with androgen-independent prostate cancer. There is a need for chemopreventive products targeting middle-aged men. This is because treatment results are not sustainable for the health system, which does not cover all the population. Developing such a product from traditional medicinal plants would facilitate better compliance among a lot of patients as the use of traditional plants and herbs are culturally embedded into the local lifestyle.

Traditional herbal medicines are still very important for Malaysians both from a nutritional and a medicinal point of view. One of the most common herbs used in Malaysia includes *Ficus deltoidea* L. *Ficus deltoidea* is a native shrub, which belongs to the family of Moracea. The plant is characterized by the evergreen small tree or shrub and in the wild the plant can reach around 5–7 m tall. This species of plant can normally be found in southeast Asian countries including Malaysia, Indonesia, and southern Philippines. It is commonly known as “Mas Cotek” in the peninsular Malaysia and people in east Malaysia normally refer to this plant as “sempit-sempit” and “agolaran” (Berg, 2003). This plant plays an important role in traditional medicine, where different parts of the plant is used for the treatment of several conditions such as the relief of headache (fruit part), toothache (fruit part), and sores and wound (roots and leaves). Women consume the decoction of boiled leaves of *F. deltoidea* as postpartum treatment to induce the contraction of the uterus and vaginal muscles besides treating the disorders of the menstrual cycle and leucorrhoea (Burkill and Haniff, 1930).

Despite this plant species having many important applications traditionally, only few studies have been conducted to explore its potential pharmacological properties. Some reported that flavonoids are one of the phytochemical compounds that can be found in abundance in *F. deltoidea* which includes gallocatechin, epigallocatechin, catechin, gallic acids, ellagic acids, luteolin-8-C-glucoside, 4-*p*-coumaroylquinic acid, orientin, vitexin, isovitexin, rutin, quercetin, and naringenin (Bunawan et al., 2014; Ramamurthy et al., 2014). The presence of these phytochemicals gives its yellow pigmentation, and many studies established that any herbs containing flavonoids could have the ability to acts as anti inflammatory, anti allergy, anti cancer and anti microbial agents, thus explaining how the plant is able to protect itself from the damaging acts of insects and microorganism (Abdullah et al., 2009; Uyub et al., 2010; Akhir et al., 2011; Zakaria et al., 2012).

Flavonoids such as epigallocatechin has been reported to have the ability to inhibit the growth of prostate cancer cells, PC3 (Albrecht et al., 2008). Zhou et al. (2009) has reported that vitexin showed cytotoxic effect on breast, ovarian and prostate cancer cells by inducing apoptosis with the cleavage of PARP protein, up-regulation of Bax and downregulation of Bcl-2. Gallic acid is identified as the major anticancer compound in *T. sinensis* leaf extract. Studies conducted using this extract have shown that gallic acid is cytotoxic against DU145 prostate cancer cells through generation of reactive oxygen species (ROS). It is also capable of blocking the growth of DU145 cells at G2/M phases by activating Chk1 and Chk2 and inhibiting Cdc25C and Cdc2 (Chen et al., 2009). Natural antioxidant such as ellagic acid has been reported to have anti-proliferative and pro-differentiation properties against prostate cancer cells by decreasing eicosanoid synthesis and downregulating the heme oxygenase system in prostate cancer cells (Vanella et al., 2013). Rutin, quercetin, and orientin have been reported to have anticancer properties by inducing apoptosis in murine leukemia WEHI-3 cells (rutin) (Lin et al., 2012), human lung cancer cell line A-549 (quercetin) (Zheng et al., 2012), and human cervical carcinoma cells, HeLa (orientin) (Guo et al., 2014). *Ficus* species that are reported to contain phenanthroindolizidine alkaloids and a series of triterpenoids with C-28 carboxylic acid functional groups demonstrate very strong cytotoxic compounds. For example, triterpenoids which were isolated from the aerial roots of *Ficus microcarpa* demonstrated cytotoxicity in three human cancer cell lines including HONE-1 nasopharyngeal carcinoma cells, KB oral epidermoid carcinoma cells, and HT29 colorectal carcinoma cells with IC50 values from 4.0 to 9.4 μM (Chiang and Kuo, 2002; Chiang et al., 2005). Since all these active phytochemicals were reported to be available in *F. deltoidea* L. (Bunawan et al., 2014), the plant could play a vital role in the inhibition of prostate cancer cells. With this in mind, the main aim of this research is to investigate both the cytotoxic effect and pro-apoptotic activities of *F. deltoidea* L., identify the bioactive compounds, and characterize the main mechanisms of the cytotoxic activity.

## Materials and Methods

### Chemicals and Reagents

#### Plant Extraction, Fractionation, and Isolation

Water, *n*-hexane (95%), ethyl acetate (>99.7%), methanol (>99.9%), and benzene-d_6_ (99.6% atom) were purchased from Sigma–Aldrich. Chloroform (99%, stabilized with amylene), acetic acid (glacial) analytical reagent grade were purchased from Fisher Scientific. Formic acid 98% was purchased from Rectapur^®^ VWR. Sephadex LH-20 was purchased from Sigma–Aldrich. TLC Silica gel 60 F_254_, was purchased from Merck KGaG, 64271 Darmstadt.

#### Cell Culture

The following tumor cell lines were used: the PC-3 cell line (ATCC Number: CRL-1435^TM^) was kindly provided by Dr. Cyrill Bussy (Centre for Drug Delivery Research, UCL School of Pharmacy, United Kingdom), the LNCaP clone FGC cell line (ATCC Number: CRL-1740^TM^) was purchased from Sigma–Aldrich, United Kingdom, and was obtained from the American Type Culture Collection (ATCC). Human Dermal Fibroblasts, adult (HDFa) catalog number C-013-5C was kindly provided by Dr. George Pasparakis (Department of Pharmaceutics, UCL School of Pharmacy). Antibiotic-antimycotic, penicillin-streptomycin, trypsin-EDTA (0.25% trpysin, EDTA 4Na), phosphate-buffered salines (PBSs), fetal bovine serum (FBS), and Eagle’s Minimum Essential Medium (EMEM) were purchased from GIBCO^®^, United Kingdom (UK). RPMI 1640 with L-glutamine was purchased from Lonza, United Kingdom. All other chemicals were obtained from Sigma–Aldrich^®^, United Kingdom, unless stated otherwise.

#### Cell Viability Assays

Sulphorhodamine B (SRB) (3-[4,5-dimethylthiazol-2-yl]-2,5 diphenyl tetrazolium bromide) (MTT), trichloroacetic acid (TCA), glacial acetic acid, 96% ethanol, isopropanol and tris base were from Sigma–Aldrich.

#### Apoptosis Assays

Caspase-Glo^®^ 3/7 detection kit and DeadEnd^TM^ Fluorometric TUNEL System were purchased from Promega. 4′,6-Diamidino-2-phenylindole (DAPI) for nucleic acid staining was from Sigma–Aldrich. Annexin-V FITC kit was from Miltenyi Biotec. MitoProbe^TM^ JC-1 Assay Kit was purchased from ThermoFisher Scientific.

#### Cell Migration and Invasion Assay

Oris^TM^ 96-well cell migration assay kit was from Platypus Technologies and CytoSelect Cell Migration Assay kit was purchased from Cell Biolab, Inc. CellTracker^TM^ Green was from Life Technologies. Cytoselect 24-well cell invasion assay kit was purchased from Cell Biolab, Inc.

#### Cell Cycle Analysis

Propidium iodide (PI) solution was purchased from Miltenyi Biotec.

#### mRNA Gene Expression Assay

Oligonucleotide primers were custom-synthesized by Primerdesign, Ltd. RNeasy^®^ Plus Mini, gDNA eliminator spin column, and Omniscript^®^ Reverse Transcription kit were purchased from Qiagen *PrecisionFAST^TM^* MasterMix with SYBR Green was obtained from Primerdesign, Ltd. DMSO was purchased from Sigma–Aldrich.

### Plant Materials Extraction and Fractionation

Certified dried plant materials of *F. deltoidea* var. *angustifolia* (FD1) and *F. deltoidea* var. *deltoidea* (FD2) were obtained from dedicated farms in Johor, Malaysia. Samples were processed using a laboratory scale mill until fine powder was produced. Vouchers for each herbal drug were deposited at the Institute of Bioproduct Development. The air-dried and powdered parts of different amount of plants (±50 g) were extracted with methanol for 72 h via a maceration process. The crude extracts were obtained after the evaporation of the methanol to complete dryness under reduced pressure at 40°C. The crude methanol extract was re-dissolved in 90% methanol and partitioned into *n*-hexane (FD1h, FD2h) chloroform (FD1c, FD2c) and aqueous extracts (FD1a, FD2a).

A sample (500 mg) of the crude chloroform extract was suspended in 2 mL of absolute methanol and applied onto a chromatographic column (2.3 cm × 40 cm) packed with sephadex LH-20 (Sigma–Aldrich^®^, United Kingdom) and equilibrated with absolute methanol. The column was exhaustively washed with absolute methanol at a flow rate of 60 mL/h. 6 mL fractions were collected using Retriever^®^ II fraction collector. 140 fractions were collected for each plant extracts.

### TLC Analysis

All fractions were examined by a thin layer chromatography (TLC) methodology on silica gel plates (TLC Silica gel 60 F_254_, Merck KGaG, 64271 Darmstadt) using (a) Chloroform-Methanol (80:20, v/v) or (b) Toulene- Ethyl Acetate-Acetic Acid (80:18:2, v/v/v) as mobile phase. The bands were visualized at white light, 254 and 366 nm wavelengths before and after derivatization with anisaldehyde using CAMAG TLC Visualizer. Eluates were then pooled into major fractions based on the TLC profiles. After evaporation of methanol, the samples were left to air-dried and residues weighed.

### Microfraction Collection Using HPLC-DAD (High Performance Liquid Chromatography-Diode Array Detector)

High performance liquid chromatography-diode array detector (HPLC-DAD) chromatograms were obtained on an Agilent 1200 series HPLC system (Agilent, United States). The stationary phase was Agilent C18 column (250 mm × 4.6 mm id, 5 μm). The microfractions were collected using Agilent 1200 series fraction collector. The data was collected and processed with the Agilent OpenLab CDS Chemstation Edition software (Agilent).

### Ultra High Performance Liquid Chromatography (UHPLC) and Mass Spectrometry (MS) Analysis

The UV (200–500 nm) and ELSD profiles were acquired to provide indication of the type of compound and a quantitative estimation of each components presence in the active fractions respectively. This was performed on Waters Acquity UHPLC system consisted of Solvent Manager, Sample Manager, Photodiode Array Detector (Waters, Milford, MA, United States) and SEDEX Model 85 LT-ELSD detector (SEDERE, Olivet, France). Data acquisition and processing was carried out using Pro Empower 3 software (Waters, Milford, MA, United States). The accurate mass measurement was acquired on Waters Acquity UHPLC system coupled with a Waters Micromass LCT Premier Time-of-Flight mass spectrometer (Waters, Milford, MA, United States) and controlled by Mass Lynx 4.1 software. Sample was ionized using electrospray ionization source which was optimized as follows: capillary voltage 2400 V, cone voltage 40 V, dessolvation temperature 330°C, source temperature 120°C, cone gas flow 20 L/h, dessolvation gas flow 700 L/h and multichannel plate detector voltage at 2450 V. The mass accuracy was calibrated for a mass range of 100–1000 using sodium formate to a mass error < 2 ppm. Leucine-enkaphalin (0.25 ppm with 0.1% formic acid) was used as internal reference and infused through the LockSpray probe at a flowrate of 7 uL/min using a dedicated LC pump (Shidmazu LC-10ADvp, Duisburg, Germany). Two microliter of 5 mg/mL sample was injected in the partial loop with needle overfill mode. The protocol for the UV, ELSD, and MS profiling was established on a C18 column (Waters Acquity UPLC BEH C18, 150 mm × 2.1 mm, 1.7 μm) that was protected with a pre-column (Waters VanGuard BEH C18, 1.7 μm). Column and sample temperature was fixed at 40 and 10°C respectively. The mobile phase consisted of water (solvent A) and acetonitrile (solvent B), each containing 0.1% formic acid (v/v). A generic 50 min gradient at a flowrate of 0.46 mL/min was applied as follows: 0–30 min, 5–95% B; 30–40 min, 95% B; 40–40.20 min, 95–5% B and end at 50 min isocratic step for column equilibration to initial conditions. The acquired MS1 raw data was converted to mzXml format using Proteowizard^1^ and imported to an open source software, MzMine 2.11^2^. Peak list was generated using 2D GridMass, followed by deisotoped step and peak alignment. Identification was performed based on a custom database created from Dictionary of Natural Products (DNPs on DVD, Windows 7 version 6.1).

### Cells Culture

Both PC-3 and LNCaP cell lines were grown in a cell culture flask (Nunc), surface area 75 cm^2^ and maintained in RPMI-1640 (Roswell Park Memorial Institute medium) (Lonza, BE12-702F) containing L-glutamine. The media was supplemented with 10% of heat-inactivated FBS (Gibco^®^, 10500-064) and 1% penicillin-streptomycin antibiotics containing 10000 Units/ml of penicillin and 10000 μg/ml streptomycin (Gibco^®^, 15140-122) to prevent bacterial growth. HDFa cell line was grown in a cell culture flask (Nunc), surface area 75 cm^2^ and maintained in DMEM (Dulbecco’s Modified Eagle Medium) containing L-glutamine and high glucose (Gibco^®^ 11965092). The media was supplemented with 10% of heat-inactivated FBS (Gibco^®^, 10500-064), 1% 100X NEAA (non-essential amino acids, Gibco^®^ 11140035), and 0.1% of both 10 mg/mL Gentamicin solution 1000X (Gibco^®^ 15710049) and 250 ug/mL Amphotericin B solution 1000X (Gibco^®^ 15290018) to prevent bacterial growth. All cells were maintained at 37°C in a humidified atmosphere of 5% CO_2_. The prepared media was used to grow and seed the cells in a 96-well plate (Nunclon^TM^) for cellular based assays and for plant extracts as well as fractions dilution.

### Cell Viability Assays

The SRB and MTT assays were performed as previously described (Vichai and Kirtikara, 2006; Houghton et al., 2007).

### Cells Morphology Analysis

PC3 and LNCaP cells were seeded in 12 well plates and left to attach and proliferate for 48 h of incubation time. Then the plant extracts were added, the morphology and the population of the cells were monitored using an EVOS^®^ FL Imaging System at 24, 48, and 72 h.

### Apoptosis Detection Assays

#### Caspases 3/7 Activity

In this study, the apoptosis induced by plant extracts was determined by measuring the activity of caspases 3 and 7 using an apoptosis detection kit (Promega, G8091). The Caspase-Glo^®^ 3/7 assay was performed according to the manufacturer’s protocol with slight modifications. PC-3 and LNCaP cells were seeded in 96 well plate at 2.5 × 10^3^. The cells were left for 24 h to allow cells attachment. After 24 h the cells were treated with extracts, fractions, vehicle control (DMSO), Paclitaxel and a blank (cell-free medium) for 48 h. 100 μl of Caspase-Glo^®^ 3/7 reagent was added to each wells after 48 h incubation period and mixed gently for 30 s. Then, the plate was incubated for 1 h at room temperature. After the completion of the incubation period, the luminescence of each plant extracts was measured using a plate-reading luminometer (Tecan Infinite^®^ M200). The assay was performed independently in triplicate and the results were calculated using the following equation:

RLU=Lu⁢minescence⁢ (samples)−Lu⁢minescence⁢ (blank)

#### Annexin V-FITC and Propidium Iodide Staining

PC3 and LNCaP cells were seeded in 12 well plates and incubated for 48 h. After incubation, plant extracts and Paclitaxel (positive control) were added and further incubated for 6 h at 37°C in a 5% CO_2_ atmosphere. The cells were then washed with PBS and detached with 0.25% Trypsin-EDTA solution. The cells were then resuspended in 100 μL of 1X binding buffer and 10 μL of Annexin V-FITC were added. The mixture was mix carefully and incubated for 15 min in the dark at room temperature. After 15 min, the cells were washed again with 1 mL of 1X binding buffer and centrifuged at 300 × *g* for 10 min. Subsequently, the cells were resuspended in 500 μL of 1X binding buffer and PI solution were added prior to flowcytometry (MACSQuant^®^ Analyzer 10) analysis.

#### Determination of Mitochondrial Membrane Potential (MMP)

The change in mitochondrial transmembrane potential (Δψ_m_) induced by the active extracts of the plants in prostate cancer cell line (PC3) was determined by using flowcytometry technique with the appropriate fluorescent probe known as JC-1 (Isenberg and Klaunig, 2000). 1 × 10^6^ cells/ml of PC3 was seeded into 6 well plate and incubated for 48 h. After incubation, plant extracts and CCCP (positive control) were added and further incubated for 6 h at 37°C in a 5% CO_2_ atmosphere. The treated cells were then labeled with 2 μM of JC-1 for 15 min at 37°C and washed with warm PBS. The cells were analyzed on a flowcytometer (MACSQuant^®^ Analyzer 10) with 488 nm with 530- and 585-nm pass emission filters. CCCP-treated cells (10 μM) were taken as positive controls.

#### Terminal Deoxynucleotidyl Transferase-Mediated Biotin dUTP Nick End Labeling Assay

Apoptotic cells were detected by using a technique called an *in situ* end labeling of the 3′-OH end of the DNA fragments generated by apoptosis-associated endonucleases. This was analyzed using the Dead End apoptosis detection kit (dUTP Nick End Labeling, TUNEL assay) acquired from Promega (Madison, WI, United States). Prostate cancer cells were grown in LabTek II chamber slide and treated with plant extracts for 72 h. The cells were then washed in PBS and 4% paraformaldehyde solution was used as fixation agent by immersing the slides in the solution for 25 min. All the steps were performed at room temperature, unless otherwise specified. Following the fixation step, the cells were then washed twice by immersing them in fresh PBS for 5 min. Cells were permeabilised using 0.1% Triton X-100 solution in PBS for 5 min, washed twice in PBS, and then covered with 100 μL of equilibration buffer and kept for 5–10 min. The equilibrated areas were blotted around with tissue paper, and 50 μL of terminal deoxynucleotidyl transferase (TdT) reaction mix was added to the sections on the slide and were then incubated at 37°C for 60 min inside a humidified chamber for the end-labeling reaction to occur. The slides were then immediately immersed in 2X sodium chloride–sodium citrate buffer for 15 min in order to terminate the reactions. Following that step, all slides were washed three times by immersing them in fresh PBS for 5 min to remove unincorporated biotinylated nucleotides. The slides were then immersed in a freshly prepared 1 μg/mL PI solution for 15 min at room temperature in the dark. The washing procedure was repeated after 15 min and then the slides were immediately analyze under a fluorescence microscope (EVOS^®^ FL Imaging System) using a standard fluorescein filter set to view the green fluorescein at 520 ± 20 nm and the red fluorescence of PI at 620 nm.

### *In Vitro* Cell Migration Assay

#### Oris^TM^96-Well 2D Cell Migration Assay

The *in vitro* cell migration was determined by using the Oris^TM^ 96-well cell migration assay kit (Platypus Technologies) following the manufacturer’s instruction. 5 × 10^4^ of PC3 cells were seeded in each well and left for 24 h. The stoppers that were used to create the migration zone were removed after 24 h and the cells were washed with PBS to remove any unattached cells. 100 μL of fresh media with or without the plant extracts were added to each well. The cells were allowed to migrate into the migration zone for 72 h. Cells were fluorescently stained with CellTracker^TM^ Green (Life Technologies). The seeded plate was incubated in a humidified chamber for 72 h and at various time points (24, 48, and 72 h), the fluorescent signals in the detection zone were measured using a microplate reader (Synergy^TM^ HT, BioTek) with 492 nm excitation and 517 nm emission filters.

#### Boyden Chamber 3D Migration Assay

The cell migration study was also analyzed using the CytoSelect Cell Migration Assay kit (Catalogue number CBA-100-C purchased from Cell Biolab, Inc.) (Ridley et al., 2003). This kit contains polycarbonate membrane inserts (8 μm pore size) in a 24-well plate. Under sterile conditions, the 24-well migration plate was allowed to warm up to room temperature for 10 min. Cell suspension containing 1.0 × 10^6^ cells/ml in serum-free media was prepared. Fresh media (control) and media with respected plant extracts, were added directly to individual transwell inserts with the cell suspension. Overnight serum starvation had been performed prior to running the assay. 500 μL of media containing 10% FBS was added to the lower well of the migration plate and 300 μL of the cell suspension solution was added to the inside of each insert and then incubated for 24 h in a cell culture incubator. After 24 h incubation, the media were carefully aspirated from the inside of the transwell insert. The interior part of the inserts was washed with wet cotton swab in order to remove non-migratory cells. The inserts were transferred to a clean well containing 400 μL of Cell Stain Solution (Crystal Violet dye) and incubated for 10 min at room temperature. Then, the inserts were gently washed in a beaker of water and left to air dry. Each inserts were transferred into wells containing 200 μL of Extraction Solution (10% acetic acid), incubated for 10 min at room temperature on an orbital shaker. 100 μL of each sample was transferred to a 96-well plate and was measured at 560 nm by using a microtiter plate reader (Tecan Infinite^®^ M200).

### *In Vitro* 3D Cell Invasion Assay

The cell invasion was measured using a Cytoselect 24-well cell invasion assay kit (Catalogue number CBA-100-C purchased from Cell Biolabs, Inc.). This kit included polycarbonate membrane inserts (8-μm pore size). The upper surface of the insert membrane was coated with a protein matrix isolated from Engelbreth-Holm-Swarm tumor cells. Basement membranes of Boyden chambers were rehydrated with 300 μL serum-free media, and 1 × 10^6^ cells were then seeded into the upper area of the chamber in serum-free media (control) with or without the plant extracts. Overnight serum starvation had been performed prior to running the assay. 500 μL of media containing 10% FBS was added to the lower well of the migration plate. After incubation for 48 h, the non-invading cells on the upper surface of the inserts were removed with a cotton swab and invading cells on the lower surface were stained with crystal violet Cell Stain Solution and incubated for 10 min at room temperature. Then, the inserts were gently washed in a beaker of water and left to air dry. Each inserts were transferred into wells containing 200 μL of Extraction Solution (10% acetic acid), incubated for 10 min at room temperature on an orbital shaker. 100 μL of each sample was transferred to a 96-well plate and was measured at 560 nm by using a microtiter plate reader (Tecan Infinite^®^ M200).

### Cell Cycle Distribution Study

PC3 and LNCaP cells were seeded in 6 well plates and incubated for 48 h. Plant extracts and Paclitaxel (positive control) were added after 48 h, and the cells were further incubated for 48 h at 37°C in a 5% CO_2_ atmosphere. The cells were then washed and trypsinized with 0.25% Trypsin-EDTA solution for cells detachment. Cell pellet were resuspended in 1 mL PBS and washed twice by adding 10 mL PBS. After that the cells were centrifuged at 300 × *g* for 10 min at 4°C. Once the supernatant was removed, 1 mL of ice-cold 70% ethanol was slowly added drop by drop to the cell pellet. The cells were allowed to fix in ethanol for 18 h. After 18 h, the cells were centrifuged at 500 × *g* for 10 min at 4°C. The supernatant was removed and 1 mL of staining solution containing 1 mg/mL PI and 100 Kunitz units/mL of RNase A were added to the cells and incubated for 30 min before being analyzed using flowcytometry (MACSQuant^®^ Analyzer 10).

### Real-Time RT-qPCR Analysis

#### mRNA Extraction and cDNA Synthesis

After exposing PC3 cells (5 × 10^5^ cells/well) to plant extracts, fractions and DMSO 1% for 96 h, total RNA was extracted using RNeasy^®^ Plus Mini (Qiagen) according to the manufacturer’s protocol. Samples were treated with gDNA eliminator spin column (Qiagen) to avoid genomic DNA contamination. The quantity and quality of RNA was determined by differential readings at 260 and 280 nm wavelength using Nanodrop 2000 (Thermo Scientific). The integrity of total RNA from PC3 cells was assessed by visual inspection of the two rRNAs 28 and 18 s on agarose gels. cDNA was synthesized from 1 μg of total RNA by using Omniscript^®^ Reverse Transcription kit (Qiagen) according to the manufacturer’s instruction in a final volume of 20 μL.

#### RT-qPCR Conditions and Analysis

Sequence for the primers used in this study are listed in **Table 1**. All primers used in this study were designed and obtained from Primerdesign, Ltd. The RT-qPCR was carried out in 96-well plates using a PikoReal^TM^ Real-Time PCR detection System (Thermo Fisher Scientific). Each well contained a final reaction volume of 20 μL (10 μL of *PrecisionFAST^TM^* MasterMix with SYBR Green, 5 μL of cDNA template diluted appropriately, 1 μL of resuspended primer mix at a final concentration of 300 nM and 4 μL of RNase/DNase free distilled water). PCR reaction was performed using the following conditions: initial denaturation at 95°C for 2 min, then 40 cycles of denaturation at 95°C for 15 s, corresponding annealing temperature of each genes as listed in **Table 1** for 30 s and extension at 72°C for 30 s. At the end of the run, heating the amplicon from 60 to 95°C in order to confirm the specificity of the amplification for each primer pair generated a melting curve. All RT-qPCR were run in quadruplicates. Standard curves were produced to check the PCR efficiency using a fivefold dilution series of cDNA. Efficiency (E) of primer pairs was obtained from the slope of the calibration curve generated. The relative expression was calculated on the basis of ‘delta delta Ct’ (ΔΔCt) values. Normalization of the target genes was achieved by using GAPDH as a reference gene.

**Table 1:** Sequence of primers used in RT-qPCR analysis.

Gene	Primer sequences	Annealing
		temperature (°C)
GAPDH	Sense: 5′-ATGCTGGCGCTGAGTACGTC-3′	55
	Anti-sense: 5′-GGGCAGAGAGATGATGACCCTT-3′	
BAX	Sense: 5′-ATGGAGCTGCAGAGGATGAT-3′	56.5
	Anti-sense: 5′-CAGTTGAAGTTGCCGTCAGA-3′	
BCL-2	Sense: 5′-GAGGTCACGGGGGCTAATT-3′	56.8
	Anti-sense: 5′-GAGGCTGGGCACATTTACTG-3′	
VEGFA	Sense: 5′-TGCTCTACTTCCCCAAATCACT-3′	57.6
	Anti-sense: 5′-CTCTCTGACCCCGTCTCTCT-3′	
Smac/ DIABLO	Sense: 5′-GCACAGAAATCAGAGCCTCATT-3′	56.4
	Anti-sense: 5′-TTCAATCAACGCATATGTGGTCT-3′	
CXCR4	Sense: 5′-CCAAAGAAGGATATAATGAAGTCACT-3′	56.4
	Anti-sense: 5′-GGGCTAAGGGCACAAGAGA-3′	
CXCL12	Sense: 5′-CTCCTCTTTCAACCTCAGTGATT-3′	56.8
	Anti-sense: 5′-GAGAAGCAGAAGCAAGATTAAGC-3′	
ALOX5	Sense: 5′-AAGCGATGGAGAACCTGTTCA-3′	56.8
	Anti-sense: 5′-GTCTTCCTGCCAGTGATTCATG-3′	


### Statistical Analysis

Data collected and reported in this study is expressed as a mean ± standard deviation. Statistical analysis of the data was carried out using the GraphPad Instat version 3 (GraphPad Software, Inc., La Jolla, CA, United States). The values of *P* < 0.05 were considered to be statistically significant. All experiments were conducted three times independently in triplicate. The Inhibitory concentration 50 (IC50) values were taken from the minimal experimental concentration showing 50% cell death and calculated using GraphPad Prism 5. Each samples was compared to the control group with GraphPad by using the Student’s *t*-test to determine any significant effect of the samples.

## Results

### Yield of the Plant Extracts

The yield of the plant extracts were; FD1c = 0.96%, FD1h = 0.27%, FD1a = 13.18%, FD2c = 0.65%, FD2h = 0.22%, FD2a = 13.10% (all in dry *w*/*w*).

### Cell Viability

FD1c and FD2c were the most cytotoxic extracts against both prostate cancer cell lines with IC values of 23 and 29 μg/mL respectively for PC3 and 19 and 23 μg/mL respectively for LNCaP.

### Cell Morphology

**FIGURE 1:**
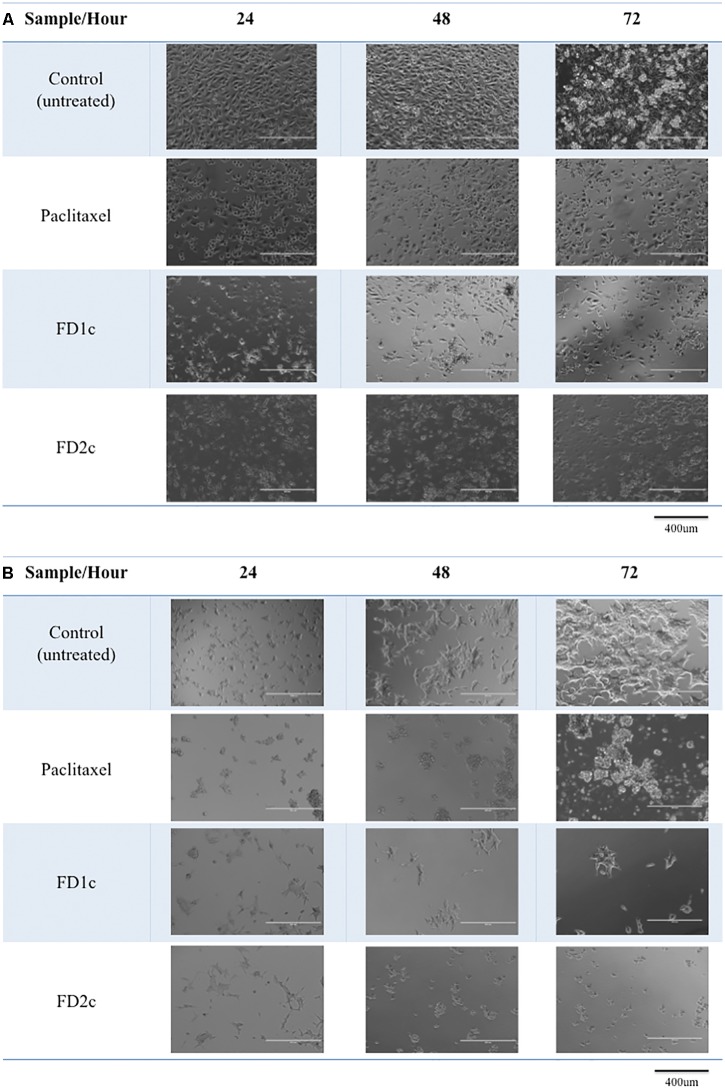
Morphological changes of **(A)** PC3 and **(B)** LNCaP cells treated with the IC50 of the plant extracts and paclitaxel (positive control) for 24, 48, and 72 h viewed under the EVOS^®^ FL Imaging System (100x magnification). Reduced in cell population was noted after 24, 48, and 72 h of treatment as compared to the control (untreated cells).

**FIGURE 2:**
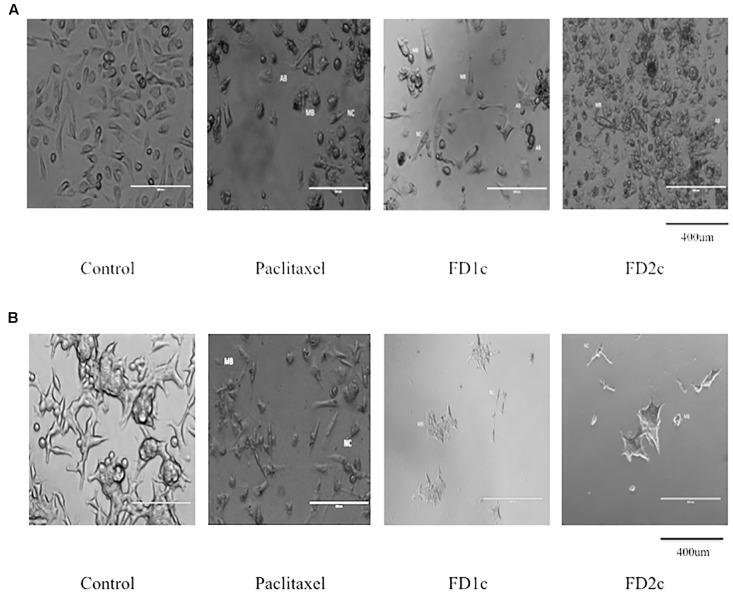
**(A)** PC3 and **(B)** LNCaP cells viewed under the EVOS^®^ FL Imaging System (200x magnification) after 72 h of treatment with the IC50 concentration of the plant extracts and paclitaxel (positive control). The cells showed characteristics of apoptosis such as the formation of apoptotic bodies (AB), membrane blebbing (MB), and nuclear compaction (NC).

In this study, the morphological changes of the prostate cancer cell lines untreated and treated with the active extracts of *F. deltoidea* L. were observed using an EVOS^®^ FL Imaging System at 24, 48, and 72 h. The characteristics of apoptosis such as cells detachment from the substratum, cell shrinkage, nuclear condensation, membrane blebbing and the formation of apoptotic bodies were detected in the treated cells (**Figures 1**, **2**). The reduction in the cell numbers is obvious in the treated populations (**Figure 1**).

### Pro-apoptotic Effect of the Active Extracts of *Ficus deltoidea* Plant

#### Stimulation of Caspases 3/7 Activity

**FIGURE 3:**
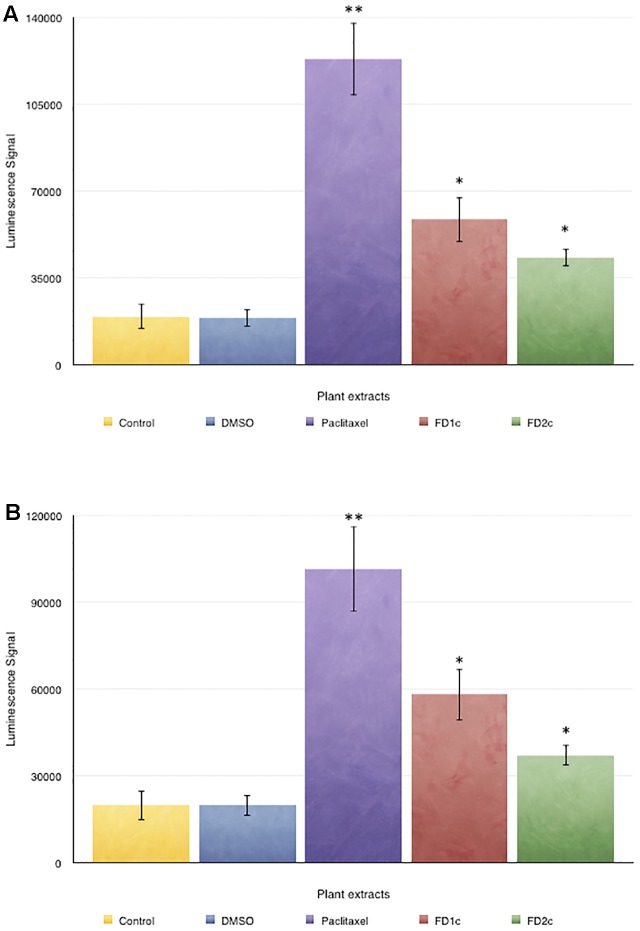
Caspase 3/7 activity in **(A)** PC3 and **(B)** LNCaP cells treated with the active extracts of *Ficus deltoidea* L. for 72 h. The y-axis shows the luminescent signal subtracted from a blank, which is proportional to the caspases activity. The error bars display the standard error of mean (SEM) obtained from three independent experiments. Significance compared to control, ^∗^*P* < 0.05, ^∗∗^*P* < 0.01 as determined by un-paired *t*-test.

Both LNCaP and PC3 (**Figure 3**) cells treated with FD1c and FD2c extracts showed significance increase (*P* < 0.01) in the activity of caspases 3 and 7 when compared to the untreated cells.

#### Detection of Apoptotic Cells Using Annexin-V FITC Staining

**FIGURE 4:**
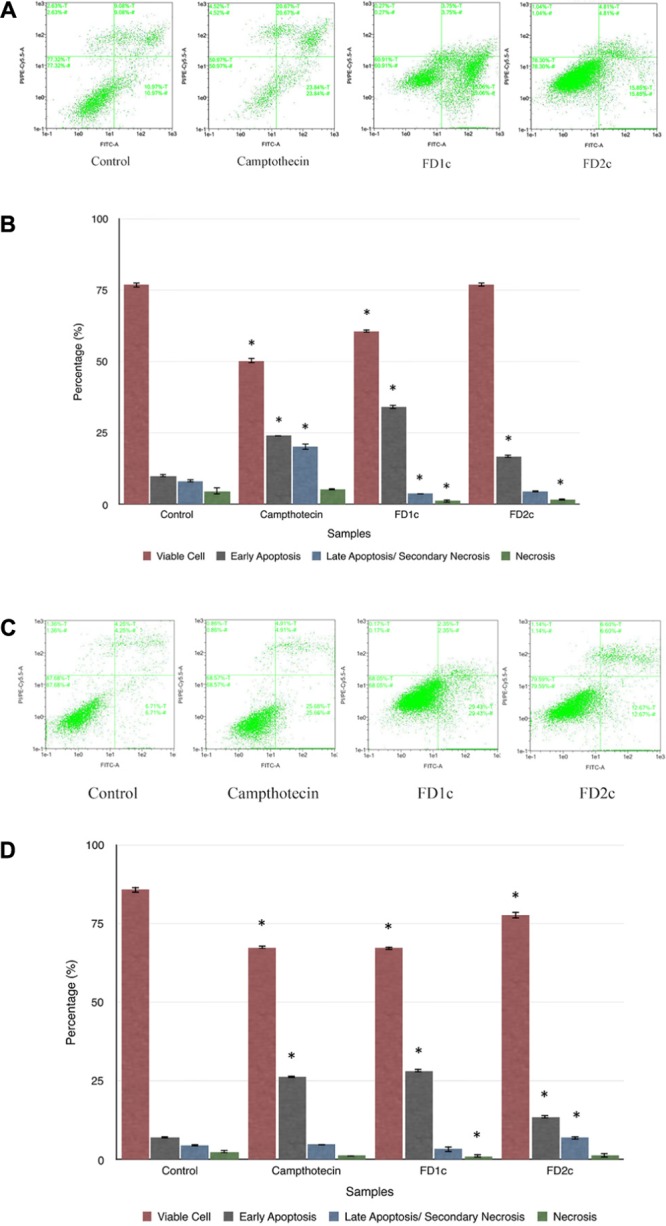
The dot plots **(A)** and the percentage (%) of cell distribution **(B)** of LNCaP cells and the dot plots **(C)** and the percentage (%) of cell distribution **(D)** of PC3 cells after 6 h treatment with the active extracts of *F. deltoidea* L. as determined by Annexin V-FITC and PI staining. The cells were treated with different active extracts for 6 h. ^∗^*P* < 0.05 as compared to the untreated cells. Results shown are representative of three independent experiments.

The results summarized in **Figure 4** show that there was a significant increase (*P* < 0.05) of the population of LNCaP cells in the early apoptosis phase after treatment with *F. deltoidea* chloroform extract (FD1c: 35%, FD2c: 16%) when compared to the untreated cells (11%). PC3 cells showed a similar trend (FD1c: 29%, FD2c:13%) (*P* < 0.05) when compared to the untreated cells (7%).

#### MMP Depolarization in PC3 Cell Line Treated with the Active Plant Extracts

**FIGURE 5:**
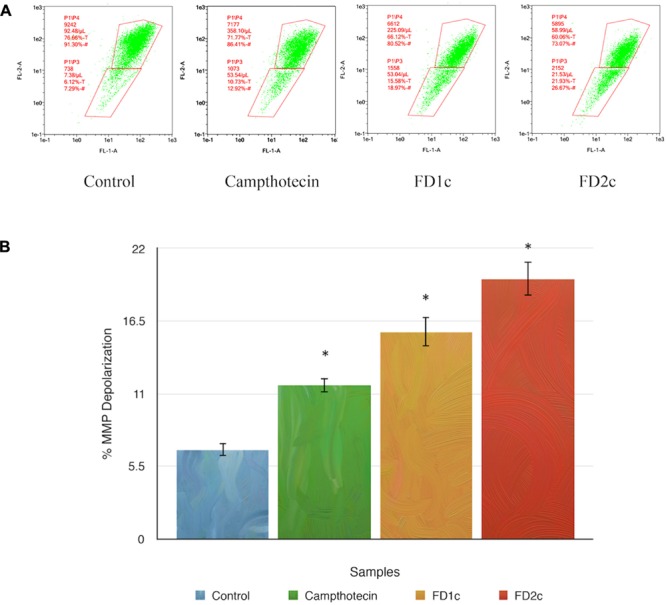
Depolarization of mitochondrial membrane potential (MMP) of PC3 prostate cancer cell lines was induced by the active extracts of *F. deltoidea* L. PC3 cell lines were treated with the IC50 of FD1c and FD2c for 6 h. **(A)** Representative MMP profiles of flow cytometry for active plant extracts-treated PC3 cells. **(B)** Quantification analysis of percentage MMP intensity in **(A)**. Data means ± SEMs (*n* = 3). ^∗^*P* < 0.05, ^∗∗^*P* < 0.01 and against control.

Mitochondrial changes, including variations in mitochondrial membrane potential (MMP) (Δψm), are the key events during drug-mediated apoptosis (Zamzami et al., 1995). One of the distinctive features of the early stage of apoptosis is the disruption of mitochondria functionality, which includes changes in the MMP and alterations to the oxidation-reduction potential of the mitochondria. **Figure 5** shows that both FD1c and FD2c increased the percentage of MMP depolarization significantly in PC3 cell lines. This could suggest that the active plant extracts induce cell death in human prostate cancer cells with the involvement of mitochondrial membrane depolarization.

#### Nuclear DNA Fragmentation

**FIGURE 6:**
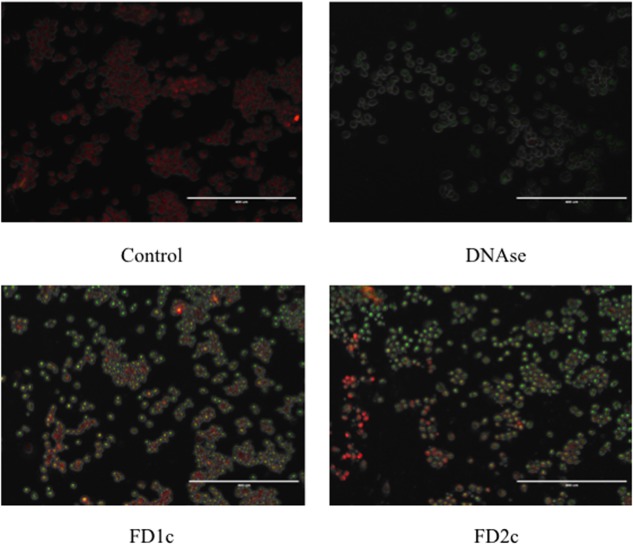
PC3 cells treated with FD1c and FD2c extracts and DNAse (positive control) and stained with DeadEnd^TM^ Flurometric TUNEL system after 72 h. Red fluorescence indicated healthy cells while green fluorescence showed fragmented nuclear DNA.

**Figure 6** shows that after 72 h treatment with active plant extracts, more localized green fluorescence (fluorescein-12-dUTP) color can be detected in the PC3-treated cells as compared to the control (untreated cells). The localized green fluorescence label can only be detected in apoptotic cells while the red PI stain will label both apoptotic and non-apoptotic cells. These findings provide more evidence that both FD1c and FD2c induce prostate cancer cell death via apoptosis.

#### Modulation of Apoptotic-Related mRNA Gene Expressions

**FIGURE 7:**
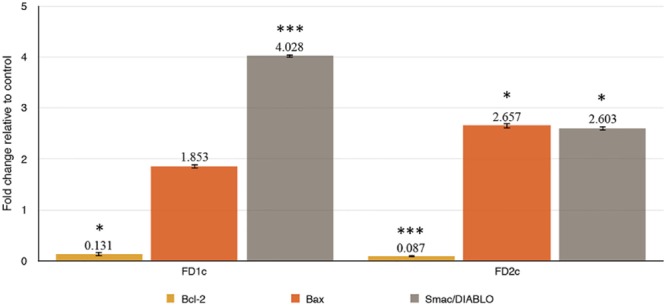
(a) Bax, (b) Bcl-2, (c) Smac/DIABLO mRNA gene expressions in PC3 cells treated with MNTC of FD1c and FD2c after 96 h. The genes expressions were determined as described in RT-qPCR Conditions and Analysis. Data are mean ± SD; *n* = 4 experiments. ^∗^*P* < 0.05, ^∗∗∗^*P* < 0.001.

Results in **Figure 7** show that FD1c and FD2c were able to increase the expression of Bax (FD1c: twofold, FD2c: twofold) and Smac/DIABLO (FD1c: fourfold, FD2c: twofold) in a significant manner. Both FD1c and FD2c also significantly down-regulated the expression of Bcl-2 by 7 and 12-fold respectively. However, no significant effect was observed for both FD1c and FD2c for the expression of ALOX5 gene, which codes for the 5-LOX protein (Data presented as Supplementary Materials). 5-LOX expression and activity are fundamental for PC3 survival (Ghosh and Myers, 1997; Werz and Steinhilber, 2006). These findings suggest that both FD1c and FD2c increase the expression of pro-apoptotic genes and reduce the expression of anti-apoptotic genes, which could lead to the increased production of pro-apoptotic proteins and reduced production of anti-apoptotic proteins and ultimately inducing PC3 cells death via apoptosis.

### Cell Cycle Arrest at G2M Phase

**FIGURE 8:**
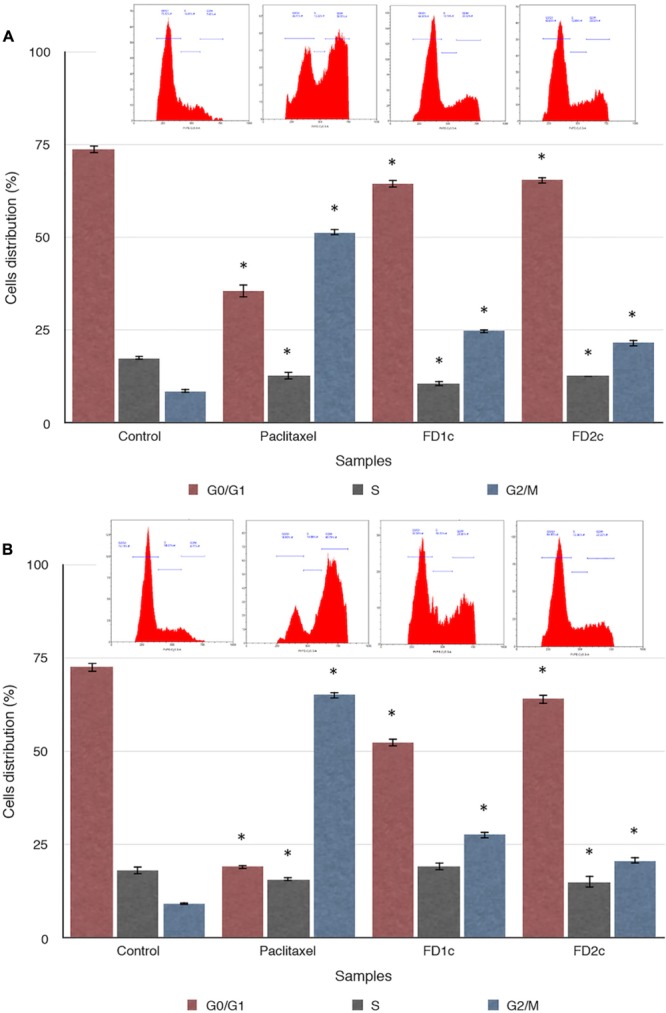
Effect of the active extracts of *F. deltoidea* L. on the cell cycle of **(A)** PC3 and **(B)** LNCaP cells analyzed by measuring DNA content using flow cytometer. The cells were treated with the IC50 of the active extracts. ^∗^*P* < 0.05 as compared to the untreated cells. Paclitaxel treatment was used as positive control. Results shown are representatives of three independent experiments.

There was a significant increase (*P* < 0.05) in the population of PC3 cells at the G2M phase as compared to the untreated cells when treated with the IC50 of both FD1c and FD2c extracts (**Figure 8A**). The same trend was observed when the LNCaP cells were treated with the IC50 of both FD1c and FD2c (**Figure 8B**). In addition, treatment with the IC50 of both plant extracts also cause a significant (*P* < 0.05) decrease in the population of both LNCaP and PC3 cells at G0/G1 phase while the percentages of cell population at the S phase remains almost the same for both cell lines.

### Inhibition of PC3 Cells Migration

**FIGURE 9:**
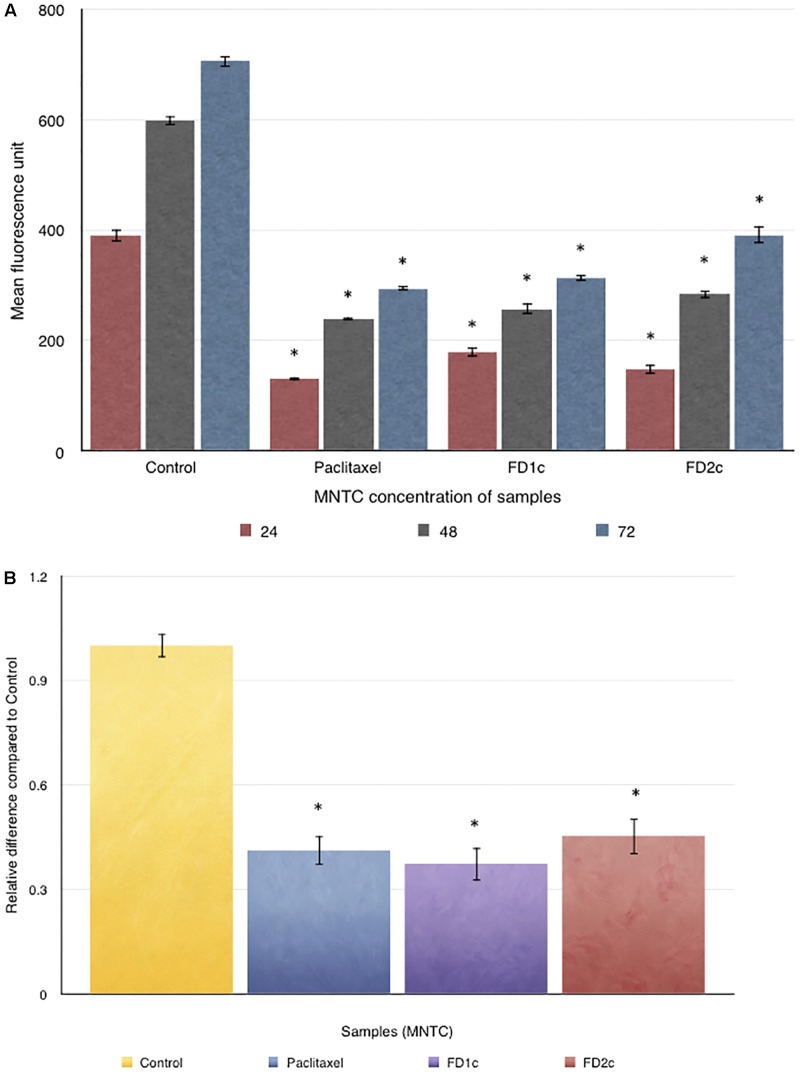
**(A)** Oris^TM^Cell Migration analysis: 5 × 10^4^ of PC3 cells were seeded per well and allowed to adhere. Stoppers were then removed and both FD1c and FD2c were added to the wells, and the plate was incubated to permit cell migration. The cells were labeled with CellTracker^TM^ Green and the fluorescence quantified in the detection zone using a Synergy HT BioTek plate reader. **(B)** CytoSelect Cell Migration analysis: effects of both FD1c and FD2c on the migration of the PC3 cells. PC3 cells were treated with the MNTC concentration of FD1c and FD2c, for 24 h. Results shown are representatives of three independent experiments, ^∗^*P* < 0.05, ^∗∗^*P* < 0.01 and against control as analyzed by the Student’s *t*-test.

**Figure 9A** indicates that both FD1c and FD2c extracts at MNTC concentration significantly inhibited the migration of PC3 cells. This observation also suggests that both FD1c and FD2c suppressed the migration of PC3 cells in a time-dependent manner. FD1c reduced the number of migrated cells by 50% at every time point. To further validate the results of this study, another migration study was conducted by using the trans-well migration assay method (Boyden Chamber). Results obtained from this experiment are shown in **Figure 9B**. After 24 h of treatment with FD1c and FD2c, the number of migrated PC3 cells has reduced significantly by almost 60% (FD1c) and 50% (FD2c). Thus, it seems quite likely that FD1c and FD2c, could inhibit PC3 cells migration.

### Inhibition of PC3 Cells Invasion

**FIGURE 10:**
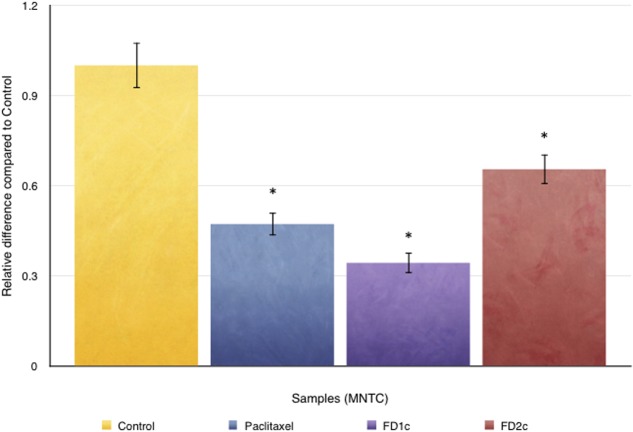
CytoSelect cell invasion analysis: effects of both FD1c and FD2c on the invasion of the PC3 cells. PC3 cells were treated with the MNTC concentration of FD1c and FD2c for 48 h. Results shown are representatives of three independent experiments, ^∗^*P* < 0.05, ^∗∗^*P* < 0.01 and against control as analyzed by the Student’s *t*-test.

**Figure 10** shows the effect of both FD1c and FD2c on PC3 cells invasion after 48 h of treatment. Both active extracts exhibited significant inhibition on the invasion of the prostate cancer cells after 48 h of treatment. FDIc extract reduces the number of invading PC3 cells by almost threefolds while FD2c extract reduces the number of the invading PC3 cells by almost twofolds in a significant manner when compared to control (*P* < 0.05). These results indicate that both FD1c and FD2c are not only capable of inhibiting or delaying PC3 cells migration but could also prevent cells invasion in a very significant way.

### Modulation of Migration and Invasion-Related mRNA Gene Expression

Both FD1c and FD2c inhibit the mRNA gene expression of CXCL12 (**Table 2**) in a significant manner. Both FD1c and FD2c extracts reduce the expression of CXCL12 by more than 20-folds. However, only FD1c was able to increase the expression of CXCR4 by twofold significantly. FD1c down-regulates the expression of VEGF by ninefolds whereas FD2c did not show any significant effect on the mRNA gene expression. The significant inhibition of the mRNA gene expression of CXCL12 by both FD1c and FD2c and the inhibition of CXCR-4 and VEGF-A by FD1c might suggest an explanation on how these plant extracts were able to suppress both migration and invasion as reported earlier.

**Table 2:** mRNA gene expression analysis (CXCR4, CXCL12, and VEGF-A) in PC3 cells treated with MNTC of the active plant extracts of *Ficus deltoidea* after 96 h.

Sample	Gene	Fold expressions relative to Control in PC3 cells
FD1c	CXCR4	2^∗^
	CXCL12	0.01^∗∗∗^ (>20-fold)
	VEGF	0.1^∗∗∗^ (ninefold)
FD2c	CXCR4	1
	CXCL12	0.003^∗∗∗^ (>20-fold)
	VEGF	1


*The genes expressions were determined as described in RT-qPCR conditions and analysis where Control = 1. Data are mean ± SD; *n* = 4 experiments. ^∗^*P* < 0.05, ^∗∗∗^*P* < 0.001.*

### Bioguided Isolation of the Active Fraction

**FIGURE 11:**
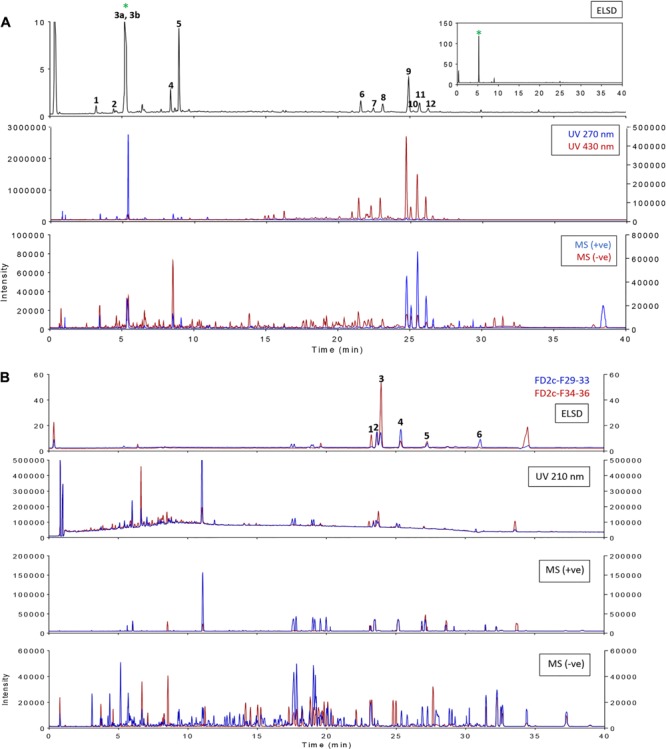
Overlay of ELSD, UV, and MS (base peaks monitoring) chromatograms for samples **(A)** FD1c F43-51 and **(B)** FD2c F29-33, FD2c F34-36.

FD1c and FD2c were chosen for further fractionation based on the IC50 values. 140 fractions were collected for each plant extracts and each fractions were examined by a TLC. Based on the TLC profiles of FD1c and FD2c (Data presented as Supplementary Materials) the eluates were then pooled into 13 major fractions (FD1c: fractions 20–23, fractions 24–30, fractions 31–36, fractions 37–42, fractions 43–51, FD2c: fraction 16, fractions 17–22, fractions 23–26, fractions 27–28, fractions 29–33, fractions 34–36, fractions 37–39, fractions 40–45). These fractions were subjected to another cytotoxicity study with PC3 cell lines to determine their activity. Three fractions were observed to have better efficacy than their respective plant extracts as their IC50 values for each fractions were significantly lower than their respective plant extracts before further fractionation process being done (*P* < 0.05). These fractions were FD1c F43-51, FD2c F29-33, and FD2c F34-36. The UHPLC-MS-ELSD-PDA profiles are shown in **Figure 11A**. The shifts for the retention time (*t*_R_) of the 12 peaks in this chromatogram are due to the sequential four-channel detection system. The universal ELSD detection method provides accurate data in accessing the relative content of individual compounds in a mixture that may not be UV active (Yang et al., 2014). Peaks 3a and 3b with *t*_R_ 5.72 min were present in the highest concentration, which accounts for 75.27% of the total mass. The positive and negative-ion ESIMS detection procedures showed different sensitivities (see **Table 3A**), with the positive mode producing a strong total ion chromatogram (TIC) for all the compounds. Therefore, the positive ESIMS and UV data were used to determine the structural information of the compounds present. Peaks 3a and 3b gave the same accurate mass of 433 and were dereplicated as Vitexin and Isovitexin respectively. These two compounds are known constituents of *F. deltoidea* (Bunawan et al., 2014). Peak 5 with *t*_R_ 9.46 min was also detected with a good concentration with 8.22% of total mass and it was dereplicated as Brosimacutin. The other 10 compounds (Compounds 1, 2, 4, and 6–12) were present in a very small concentration and thus not considered to play a major role in the cytotoxic activity of FD1c F43-51 fraction. FD2c F29-33 and FD2c F34-36 were active against PC3 cancer cell lines with IC50 values of 28 and 24 μg/mL respectively. The UHPLC-MS-ELSD-PDA chromatogram in **Figure 11B** shows six peaks. For FD2c F29-33, four major peaks were detected – peak 1 is similar to peak 3 – at *t*_R_ 24.15 (peak 2), *t*_R_ 24.42 (peak 3), *t*_R_ 25.87 (peak 4), and *t*_R_ 31.59 (peak 6). These peaks correspond to 21.07% (peak 3 + peak 1), 20.09% (peak 2), 34.40% (peak 4), and 31.59% (peak 6) of total mass respectively. ESIMS data shows that these peaks gave accurate masses of 457, 425, 427, and 443 respectively and were dereplicated as Oleanolic acid, Lupenone, Lupeol, Moretenol, and Betulin. For FD2c F34-36 fraction, two major compounds were dereplicated as Oleanolic acid – at *t*_R_ 24.42, which correspond to 73.33% (peak 1 + peak 3) of total mass – and Lupenone at *t*_R_ 24.15, and *t*_R_ 27.77 – which correspond to 18.65% of total mass. Both Oleanolic acid and Lupenone gave accurate masses of 457 and 425 respectively according to the ESIMS data (see **Table 3B**).

**Table 3A:** MS1 dereplication of FD1c F43-51 samples (*v. angustifolia*).

Peak no.	UHPLC-DAD-ELSD			UHPLC-DAD-TOF MS				Proposed identification
		
	Time (min)	% Area	PDA (UVmax)	[M+H]^+^		[M-H]^-^		
						
				Accurate mass	Molecular formula (mass error in ppm)	Accurate mass	Molecular formula (mass error in ppm)	
1	3.71	0.91	271, 325	595.1660	C_27_H_31_O_15_ (0.50)	593.1518	C_27_H_29_O_15_ (1.18)	4′,5,6,7-Tetrahydroxyflavone; 6-*O*-[α-L-Rhamnopyranosyl-(1→2)-β-D-galactopyranoside]^a^ 4′,5,6,7-Tetrahydroxyflavone; 7-Me ether, 6-*O*-[α-L-arabinopyranosyl-(1→2)-β-D-glucopyranoside]^a^ Isovitexin-7-*O*-beta-D-glucopyranoside^c^
2	4.92	0.42	270, 349	449.1080	C_21_H_21_O_11_ (0.44)	447.0935	C_21_H_19_O_11_ (0.67)	Isoorientin^d^
3a	5.72	75.27	268, 351	433.1132	C_21_H_21_O_10_ (0.69)	431.0983	C_21_H_19_O_10_ (0.0)	Vitexin^d^
3b					C_21_H_21_O_10_ (0.69)	431.0982	C_21_H_19_O_10_ (0.23)	Isovitexin^d^
4	8.88	2.20	250, 312	305.1016	C_16_H_17_O_6_ (0.98)	349.0931 [M-H+FA]^-^	C_17_H_17_O_8_ (4.01)	Aviprin^d^
5	9.46	8.22	231, 280	359.1483	C_20_H_23_O_6_ (1.7)	No signal		2′,4′-Dihydroxy-7-methoxyflavan-8-butanoic acid^b^ Brosimacutin A, Brosimacutin B, Brosimacutin M^b^ 2,2′,4,4′-Tetrahydroxy-3′-(4-hydroxy-3-methyl-2-butenyl)dihydrochalcone^b^
6	22.08	1.66	296, 369, 436	623.2503	C_35_H_35_N_4_O_7_ (0.43)	No signal		C_35_H_35_N_4_O_7_
7	22.95	0.64	295, 368, 435	623.2507	C_35_H_35_N_4_O_7_ (0.43)	No signal		C_35_H_35_N_4_O_7_
8	23.62	1.20	297, 331, 370, 436	607.2552	C_35_H_35_N_4_O_6_ (0.15)	No signal		Phaeophorbide b^c^
9	25.40	6.58	230, 277, 410	609.2717	C_35_H_37_N_4_O_6_ (1.54)	No signal		Mesophaeophorbide b^c^ 10-Hydroxy phaeophorbide a^c^
10	25.73	0.27	226, 277, 401	625.2658	C_35_H_37_N_4_O_7_ (0.19)	No signal		Purpurin 7; 3′′′-Mono-Me ester^c^ 15^2^-Hydroxylactone pheophorbide a^c^
11	26.16	1.88	230, 277, 409	609.2718	C_35_H_37_N_4_O_6_ (1.70)	No signal		Mesophaeophorbide b^c^ 10-Hydroxy phaeophorbide a^c^
12	26.77	0.76	229, 327, 410	593.2762	C_35_H_37_N_4_O_5_ (0.59)	No signal		Phaeophorbide a^c^ Phaeoporphyrin a5^c^, 10-Epimer phaeophorbide a^c^


*^a^Match with DNP (*Ficus*); ^b^match with DNP (Moraceae); ^c^match with DNP (Dicot), and ^d^match with isolated compound.*

**Table 3B:** MS1 dereplication of FD2c samples (v. *deltoidea*).

Peak no.	UHPLC-DAD-ELSD			UHPLC-DAD-TOF MS (Positive ionization)		Proposed identification
			
	Time (min)	% Area (F29-33)	% Area (F34-36)	Accurate mass (M+H)^+^	Molecular formula (mass error in ppm)	
1	23.75	1.18	10.81	439.3576	C_30_H_47_O_2_ (1.36)	Moretenolactone Bengalensinone 20-Taraxastene-3,22-dione
2	24.15	20.09	12.72	425.3784	C_30_H_49_O (1.65)	Cycloartenone^c^ Lupenone^d^
3	24.42	19.89	62.52	457.3678	C_30_H_49_O_3_ (0.43)	3α-Hydroxy-22(29)-isohopen-24-oic acid^a^ 19,20-Seco-3,19,20-ursanetrione^a^ Moruslupenoic acid A^b^ Oleanolic acid^c^
4	25.87	34.40	8.01	443.3872	C_30_H_51_O_2_ (2.48)	13(18)-Neohopene-3,24-diol; 3β-form^a^ Gmeliniin A, 21α-Hydroxytaraxasterol^a^ 20-Taraxastene-3,22-diol; (3β,22β)-form^a^ 29(20→19)-Abeo-3-hydroxy-20-lupanone; (3β,19βMe)-form^a^
5	27.77	6.50	5.93	425.3766	C_30_H_49_O (2.58)	Cycloartenone^c^ Lupenone^d^
6	31.59	17.94	Not determined	427.3924	C_30_H_51_O (2.34)	Moretenol^a^ Lupeol^a^ Rhoiptelenol^a^ 3-Serratanone^a^
				425.3760	C_30_H_49_O (3.99)	Cycloartenone^b^ Lupenone^c^


*^a^Match with DNP (*Ficus*); ^b^match with DNP (Moraceae); ^c^match with DNP (Dicot), and ^d^match with isolated compound.*

### Modulation of mRNA Gene Expression by the Active Fractions

#### Apoptosis-Related Genes Expression

FD2c F29-33 and FD2c F34-36 significantly inhibited Smac/DIABLO mRNA gene expression by five and five folds respectively. However, FD1c F43-51 did not show any significant effect on the expression of Smac/DIABLO. Therefore, these data suggest that the active fractions of the plant extracts (FD2c F29-33, and FD2c F34-36) also induced PC3 prostate cancer cells death via apoptosis as observed with their active extracts.

#### Migration-Related Genes Expression

**Table 4** shows that FD1c F43-51 has significantly down-regulated the mRNA gene expression of VEGF by fourfold, whereas both active fractions of the FD2c (FD2c F29-33 and FD2c F34-36) extract did not cause any significant changes to the expression of VEGF. Even though none of the active fractions significantly caused any changes to the expression of CXCR4, all 3 of them had caused significant inhibition to the expression of CXCL12 by more than 20-fold. These findings are similar to the previous results observed with the active extracts of the plants but with greater efficacy. Therefore, the active fractions of the plant extracts also inhibit PC3 prostate cancer cells migration and invasion through similar mode of action.

**Table 4:** mRNA gene expression analysis (VEGF-A, CXCR4, and CXCL12) in PC3 cells treated with MNTC of the active fractions of *Ficus deltoidea* after 96 h (migration-related genes).

Sample	Gene	Fold expressions relative to Control in PC3 cells
FD1c F43-51	VEGF-A	0.3^∗∗∗^ (fourfold)
	CXCR4	1
	CXCL12	0.02^∗^ (>20-fold)
FD2c F29-33	VEGF-A	1
	CXCR4	1
	CXCL12	0.005^∗∗∗^ (>20-fold)
FD2c F34-36	VEGF-A	1
	CXCR4	2
	CXCL12	0.005^∗∗∗^ (>20-fold)


*The genes expressions were determined as described in RT-qPCR conditions and analysis where Control = 1. Data are mean ± SD; *n* = 4 experiments. ^∗^*P* < 0.05, ^∗∗∗^*P* < 0.001.*

#### Chromatographic and Mass Spectrometry Analysis

MS1 dereplication is one of the first approaches in structural identification that is a common practice before MS/MS dereplication and isolation work (Alali and Tawaha, 2009). It is established by generating molecular formula from the accurate mass data and comparing it with a bibliographical database such as DNP, Universal Natural Products Database (UNPD), and MarinLit. In this study, the custom database was created from DNP by using three taxonomy filters; species, family, and type of organism (*Ficus* 272 hits; Moraceae: 1,607 hits; Dicot: 107,953 hits). In addition, the type of compound indicated by the UV spectrum data was further used to reduce the number of proposed identification and improve the accuracy of the proposed identification. As an initial attempt to investigate the composition of the active extracts, focus was given only to components that gave a reasonable ELSD signal (quantitative estimate). The corresponded mass spectrometry detection with the ELSD signals for var. *angustifolia* and var. *deltoidea* are shown in **Figures 11A,B**, respectively. The two active fractions from var. *deltoidea* seemed to be similar in composition. **Tables 3A,B** showed the dereplication results and ideally, whenever available, structural identifications were confirmed by co-chromatography with commercially available standards or compounds isolated in the lab. The var. *angustifolia* primarily consisted of flavonoid glycosides, furanocoumarin and chlorophylls derivatives while var. *deltoidea* could be characterized by triterpenoids.

### Anti-proliferative Activity of Dereplicated Compounds

Previously, LC–MS dereplication has identified isovitexin in the active fraction of FD1c and oleanolic acid, betulin, and lupeol in the active fractions of FD2c. This study was conducted to evaluate the cytotoxic activity of the individual compounds identified through LC–MS dereplication method. SRB staining assay was used for the purpose of this study. LC–MS dereplication has identified isovitexin as one of the major compound in the active fraction of FD1c, however the IC50 value for isovitexin (IC50 = 43 μg/ml) is significantly (*P* < 0.05) higher than its respective active fraction (IC50 = 12 μg/ml). Similar findings were observed with the IC50 values for oleanolic acid (IC50 = 34 μg/ml), botulin (IC50 = 47 μg/ml), and lupeol (IC50 = 36 μg/ml), which were identified through LC–MS dereplication of the active fractions of FD2c. The IC50 values for each of these compounds were significantly (*P* < 0.05) higher than their respective active fractions (FD2c F29-33 = 28 μg/ml, FD2c F34-36 = 24 μg/ml). These results indicated that the active fractions of both FD1c and FD2c were more cytotoxic against PC3 cells than each of these individual compounds. In the case of FD1c, even though isovitexin was identified as the major compounds in the active fraction, lower cytotoxic activity of the compound against PC3 cells might suggest that the other minor compounds such as brosimacutin and phaeoporphyrin might have contributed to the more potent cytotoxic activity of the active fraction. In the active fractions of FD2c, oleanolic acid, betulin, and lupeol were dereplicated as the major compounds in the active fraction. However, as mentioned earlier, none of these individual compounds were shown to have better cytotoxic activity than their respective active fractions. Once again, this could suggest that the more potent cytotoxic activity of the active fractions of FD2c might be due to the additives or synergistic effects of the compounds identified in the plant.

### Differential Cytotoxicity in Human Dermal Fibroblasts Cells (HDFa)

This study is carried out in order to investigate the effect of the active plant extracts on normal human cells. We established the Maximum Non-Toxic Concentrations of the extracts (MNTC) as the concentration allowing 80% of the cell population to grow. The three active fractions, FD1c F43-51, FD2c F29-33, and FD2c F34-36 were observed to have MNTC value of more than 200 μg/mL. This information indicates that all the active fractions of the plant show good selectivity and are only specific in inhibiting prostate cancer cell lines (Data presented as Supplementary Materials).

## Discussion

Two different cell lines have been used in this study (PC3 and LNCaP) as each cell line represent different stage of prostate cancer, the androgen-dependent and the androgen-independent prostate cancer, respectively (Shafi et al., 2013). From our investigations, all active plant extracts and their respective fractions were cytotoxic against both types of prostate cancer cell lines with lower IC50 values for PC3.

According to recent statistics provided by the Office for National Statistics (2016), approximately 90% of androgen-dependent prostate cancer are detected in the local and regional stages, therefore the cure rate is very high as nearly 100% of men diagnosed and treated at this stage will be disease free after 5 years. However, many men die as therapy eventually fails when the disease progresses to androgen-independent stage (Feldman and Feldman, 2001). This stage of prostate cancer is lethal and has the ability to progress and metastasize. At present, there is no effective therapy that can be used for men diagnosed with androgen independent prostate cancer.

Therefore, we turned our main focus to investigate the effect of the active plant extracts on PC3 cells. The active plant extracts induce apoptosis in PC3 cells via the intrinsic pathway, as evidenced by the significant activation of caspases 3 and 7. This activation is mediated -at least in part- by their ability to affect the gene expression of proteins such as Bcl-2, and Smac/DIABLO. Smac/DIABLO is a novel mitochondria-derived pro-apoptotic protein that plays an important role in sensitizing tumor cells to die by apoptosis (Du et al., 2000; Verhagen et al., 2000). It has a pro-apoptotic effect that is mediated by its interaction with inhibitor of apoptosis proteins (IAPs) and the release of effector caspases from them. The IAP protein family that includes XIAP, c-IAP1 and c-IAP2 blocks both intrinsic and extrinsic apoptotic pathways by binding to and inhibiting active caspases, thus stopping the caspase cascade (Jia et al., 2003). Smac/DIABLO functions by neutralizing the caspase-inhibitory properties of XIAP (Ekert et al., 2001). This is achieved by the displacement of XIAP from caspase 9 by Smac/DIABLO, thus overcoming the ability of XIAP to repress the activity of effector caspase, caspase-9, within the apoptosome complex (Srinivasula et al., 2001). Carson et al. (2002) have shown that Smac/DIABLO is required for mitochondrial-driven apoptosis in human multiple myeloma and prostate cancer cells. Smac peptide was also reported to enhance apoptosis induced chemo or immunotherapeutic agents in the leukemic Jurkat cell line (Guo et al., 2002), and in malignant glioma cells *in vivo* (Fulda et al., 2002). Therefore, these findings prompted scientists to develop or find small molecules that could mimic the functions of Smac/DIABLO as therapeutic agents to induce cancer cells death or increase the apoptotic effects of the chemotherapeutic agents. This study shows that both active plant extracts (FD1c and FD2c) were able to significantly increase the expression of Smac/DIABLO gene in PC3 cells, thus contributing -in part- in activating the intrinsic pathway of apoptosis. However, upon further fractionation of the active extracts, the active fraction of FD1c (FD1c F43-51) did not show any significant effect on the expression of Smac/DIABLO. This could happen due to the loss of active metabolite/s during the fractionation process. Even though FD1c F43-51 showed better efficacy than its active extracts, the loss of some bioactive component/s might lead to PC3 cell death through a different mode of action. Other active fractions showed a similar trend in activity when compared to their active extracts but with better efficacy.

In this study, we have also decided to investigate the effect of both plant extracts on the expression of ALOX5 gene. ALOX5 is a gene responsible for the production of 5-LOX enzyme in human. Ghosh and Myers (1997) reported that chemical constituents such as arachidonic acid, an omega-6, polyunsaturated fatty acid was found to stimulate prostate cancer cell growth via the 5-LOX pathway. This has been recently corroborated by Yang et al. (2012), who also point toward 12-LOX. The expression of 5-LOX is normally restricted to specified immune cells such as neutrophils, eosinophils, basophils, and macrophages whereas the vast majority of non-immune body cells do not express 5-LOX unless at the onset of certain diseases such as asthma, arthritis, psoriasis, and cancer (Fürstenberger et al., 2006; Werz and Steinhilber, 2006; Rådmark et al., 2007; Ghosh, 2008). 5-LOX plays a very important role in chemotaxis in these cells. Ghosh and Myers (1997) and Werz and Steinhilber (2006) reported that the inhibition of 5-LOX would block the production of 5-LOX metabolites and triggers apoptosis in prostate cancer cells. The expression of 5-LOX in normal prostate glands is almost undetectable, however, 5-LOX is heavily expressed in prostate tumor tissues including PC3 cell lines. However, this study shows that the active extracts of both plants (FD1c and FD2c) did not show any significant effect on the expression of ALOX5 gene. This suggests that both active extracts induced prostate cancer cells death via other pathways, which are not related to the inhibition of 5-LOX metabolites production.

This pro-apoptotic effect lead to a reduction of the cell population in the G0/G1 phase, whereas those in the G2/M phase was increased significantly when compared to the untreated PC3 cells. Cell cycle arrest specifically at the G2/M phase serves to prevent cells with damaged genomic DNA from entering the mitosis (M) phase.

Additionally, all anti-proliferative plant extracts (FD1c and FD2c) displayed significant inhibitory effects on the migration and invasion of PC3 cancer cell lines at non-toxic concentrations. Further mechanistic studies showed that the active plant extracts and their respective fractions (FD1c F43-51, FD2c F29-33, and FD2c F34-36) were able to inhibit cell motility by modulating CXCR4, CXCL12 gene expression. Increasing evidence indicate that the tumor microenvironment (including tumor-stromal cells interactions) have a crucial role in tumor initiation and progression (Guo et al., 2015). Singh et al. (2004) reported that CXCL12-CXCR4 interactions were able to modulate prostate cancer cell migration and invasion. CXCL12 is a type of chemokines that bind to and activate a family of chemokine receptors (Vindrieux et al., 2009). CXCR4 is a type of chemokine receptor that consists of seven-transmembrane receptors coupled to G protein (Ransohoff, 2009). CXCL12 and chemokine receptors CXCR4 and CXCR7, are the key factors that link between cancer cells and their microenvironment. Functional CXCR4 was reported to be expressed by prostate cancer cell lines PC3 and LNCaP, as well as normal prostate epithelial cells (PrEC). However, significantly higher levels of CXCR4 were observed in the malignant cell lines such as PC3 and LNCaP compared to the normal prostatic epithelial cells. Previous studies conducted by Singh et al. (2004) shows that the number of malignant prostate cancer cell lines that migrated in response to CXCL12 was significantly higher than for cells not exposed to CXCL12 as chemoattractant. Therefore, interruption to the level of CXCL12 and disruption to CXCL12-CXCR4 litigation could possibly lead to the inhibition of prostate cancer cells migration and invasion. All the active extract of the plants and their respective fractions were able to down-regulate the CXCL12 mRNA gene expression significantly. This might lead to the fall of the production of CXCL12 chemokines and thus inhibiting both PC3 cells migration and inhibition. However, only FD1c but not its respective fraction (FD1c F43-5) was able to have a significant effect on the expression of CXCR4.

Regarding the mRNA gene expression of VEGF, only FD1c F43-51 had shown significant inhibition to the expression of the gene. Vascular endothelial growth factor (VEGF) is known to be a critical regulator of endothelial cell migration by increasing endothelial cell permeability, stimulating proliferation, and promoting migration of phosphatidylinositol-3-kinase and the small GTPase Rac-1 (Soga et al., 2009). Apart from that, VEGF also play a very important role in the formation of new blood vessels from pre-existing capillaries and venules – a process called angiogenesis. Overexpression of VEGF is normally seen in cancer cells, as it is vital to help create the microenvironment that would promote the growth of the cells. Therefore, by reducing the expression of VEGF, the production of its protein will be decreased and thus creating a microenvironment that is not suitable for the cancer cells to migrate or invade. Despite the cytotoxic effects of all the active plant extracts and their respective fractions on prostate cancer cell lines, they showed specific selectivity when tested with normal human dermal fibroblast cells (HDFa).

The LC–MS dereplication of active fractions of *Ficus* spp. identified isovitexin in FD1c F43-51; oleanolic acid, moretenol, betulin, lupenone, and lupeol in FD2c F29-33; and oleanolic acid, and lupenone in FD2c F34-36. Both isovitexin and moretenol have been previously reported to be isolated from *F. deltoidea* (Bunawan et al., 2014). However, this is the first time that oleanolic acid, betulin, lupenone, and lupeol are identified in this species. Oleanolic acid has been identified in *Ficus microcarpa* (Ao et al., 2008), *Ficus hispida* Linn (Joseph and Raj, 2011), and *Ficus nervosa* (Chen et al., 2010). Betulin was identified in *Ficus foveolata* (Somwong et al., 2013), *Ficus racemosa* (Bopage, 2016), and *Ficus carica* L. (Oliveira et al., 2012). Lupenone was identified in *Ficus pseudopalma* (Ragasa et al., 2009), *Ficus microcarpa* (Kuo and Lin, 2004), and *Ficus sycomorus* (Ahmadu et al., 2007). While, lupeol has been previously identified in *Ficus pseudopalma* Blanco (Santiago and Mayor, 2014), *Ficus odorata* (Tsai et al., 2012), and *Ficus polita* Vahl (Kuete et al., 2011).

## Conclusion

FD1c, FD2c and the respective active fractions were able to overcome three main hallmarks of cancer in PC3 cells: (1) apoptosis by activating of the intrinsic pathway, (2) inhibition of both migration and invasion by modulating the CXCL12-CXCR4 axis, and (3) inhibiting angiogenesis by modulating VEGF-A expression. These activities can be concentrated in phytochemically well-defined active fractions containing a number of known and new (vitexin) cytotoxic compounds toward PC3 cells.

## Author Contributions

MH contributed to the experimental design and was in charge of performing and setting up all the assays involving PC3 and LNCaP cell lines and the writing of the manuscript. HY contributed by sourcing and extracting the plant material. JP contributed to the experimental design and the writing of the manuscript. AA and J-LW contributed with the dereplication of the bioactive fractions. RA and MS contributed in getting the permission to work with the plant materials and providing the assistance in securing the funding.

## Conflict of Interest Statement

The authors declare that the research was conducted in the absence of any commercial or financial relationships that could be construed as a potential conflict of interest.

## Footnotes

1 http://proteowizard.sourceforge.net/tools.shtml

2 http://sourceforge.net/projects/mzmine/

## References

[B1] Abdullah Z. Hussain K. Ismail Z. Ali R. M. (2009). Anti-inflammatory activity of standardised extracts of leaves of three varieties of *Ficus deltoidea*. *Asian J. Pharm. Clin. Res.* 1 100–105.

[B2] Ahmadu A. Agunu A. Enhimidu J. O. Magiatis P. Skaltsounis A. L. (2007). Antibacterial, anti-diarrheal activity of *Daniellia oliveri* and *Ficus sycomorus* and their constituents. *Planta Med.* 73 172 10.1055/s-2007-986953

[B3] Akhir N. A. M. Chua L. S. Majid F. A. A. Sarmidi M. R. (2011). Cytotoxicity of aqueous and ethanolic extracts of *Ficus deltoidea* on human ovarian carcinoma cell line. *Br. J. Med. Med. Res.* 1 397–409. 10.9734/BJMMR/2011/507

[B4] Alali F. Q. Tawaha K. (2009). Dereplication of bioactive constituents of the genus *Hypericum* using LC-(+,-)-ESI-MS and LC-PDA techniques: *Hypericum triquterifolium* as a case study. *Saudi Pharm. J.* 17 269–274. 10.1016/j.jsps.2009.10.002 23960710 PMC3730979

[B5] Albrecht D. S. Clubbs E. A. Ferruzzi M. Bomser J. A. (2008). Epigallocatechin-3-gallate (EGCG) inhibits PC-3 prostate cancer cell proliferation via MEK-independent ERK1/2 activation. *Chem. Biol. Interact.* 171 89–95. 10.1016/j.cbi.2007.09.001 17931610

[B6] Ao C. Li A. Elzaawely A. A. Xuan T. D. Tawata S. (2008). Evaluation of antioxidant and antibacterial activities of *Ficus microcarpa* L. fil. extract. *Food Control* 19 940–948. 10.1016/j.foodcont.2007.09.007

[B7] Berg C. (2003). Flora Malesiana precursor for the treatment of Moraceae 3: *Ficus* subgenus *Ficus*. *Blumea Biodiv. Evolut. Biogeogr. Plants* 48 529–550. 10.3767/000651903X489537

[B8] Bopage N. (2016). Investigation on wound healing activity of bark of *Ficus racemosa* and “Seetodaka” oil using scratch wound assay (SWA). *Chem. Sri Lanka* 33 ISSN 1012-8999: 18

[B9] Bunawan H. Amin N. M. Bunawan S. N. Baharum S. N. Mohd Noor N. (2014). Ficus deltoidea Jack: a review on its phytochemical and pharmacological importance. *Evid Based Complement. Alternat. Med.* 2014:902734. 10.1155/2014/902734 24772185 PMC3977116

[B10] Burkill I. H. Haniff M. (1930). Malay village medicine: prescriptions collected. *Gard. Bull. Strait Settlements* 6 176–177.

[B11] Cancer Research UK (2016). *Prostate Cancer Statistics.* Available at: http://www.cancerresearchuk.org/health-professional/cancer-statistics/statistics-by-cancer-type/prostate-cancer-heading-Zero

[B12] Carson J. P. Behnam M. Sutton J. N. Du C. Wang X. Hunt D. F. (2002). Smac is required for cytochrome c-induced apoptosis in prostate cancer LNCaP cells. *Cancer Res.* 62 18–23. 11782351

[B13] Chen H. M. Wu Y. C. Chia Y. C. Chang F. R. Hsu H. K. Hsieh Y. C. (2009). Gallic acid, a major component of *Toona sinensis* leaf extracts, contains a ROS-mediated anti-cancer activity in human prostate cancer cells. *Cancer Lett.* 286 161–171. 10.1016/j.canlet.2009.05.040 19589639

[B14] Chen L. W. Cheng M. J. Peng C. F. Chen I. S. (2010). Secondary metabolites and antimycobacterial activities from the roots of *Ficus nervosa*. *Chem. Biodiver.* 7 1814–1821. 10.1002/cbdv.200900227 20658670

[B15] Chiang Y. M. Chang J. Y. Kuo C. C. Chang C. Y. Kuo Y. H. (2005). Cytotoxic triterpenes from the aerial roots of *Ficus microcarpa*. *Phytochemistry* 66 495–501. 10.1016/j.phytochem.2004.12.026 15694457

[B16] Chiang Y.-M. Kuo Y.-H. (2002). Novel Triterpenoids from the Aerial Roots of *Ficus microcarpa*. *J. Organ. Chem.* 67 7656–7661. 10.1021/jo020262e 12398486

[B17] Du C. Fang M. Li Y. Li L. Wang X. (2000). Smac, a mitochondrial protein that promotes cytochrome c–dependent caspase activation by eliminating IAP inhibition. *Cell* 102 33–42. 10.1016/S0092-8674(00)00008-8 10929711

[B18] Ekert P. G. Silke J. Hawkins C. J. Verhagen A. M. Vaux D. L. (2001). DIABLO promotes apoptosis by removing MIHA/XIAP from processed caspase 9. *J. Cell Biol.* 152 483–490. 10.1083/jcb.152.3.483 11157976 PMC2195997

[B19] Feldman B. J. Feldman D. (2001). The development of androgen-independent prostate cancer. *Nat. Rev. Cancer* 1 34–45. 10.1038/35094009 11900250

[B20] Ferlay J. Soerjomataram I. Ervik M. Dikshit R. Eser S. Mathers C. (2013). *GLOBOCAN 2012 v1.0 Cancer Incidence and Mortality Worldwide: IARC CancerBase No. 11 [Internet].* Lyon: International Agency for Research on Cancer.

[B21] Fulda S. Wick W. Weller M. Debatin K. M. (2002). Smac agonists sensitize for Apo2L/TRAIL-or anticancer drug-induced apoptosis and induce regression of malignant glioma in vivo. *Nat. Med.* 8 808–815. 10.1038/nm735 12118245

[B22] Fürstenberger G. Krieg P. Müller-Decker K. Habenicht A. J. (2006). What are cyclooxygenases and lipoxygenases doing in the driver’s seat of carcinogenesis? *Int. J. Cancer* 119 2247–2254. 16921484 10.1002/ijc.22153

[B23] Ghosh J. (2008). Targeting 5-lipoxygenase for prevention and treatment of cancer. *Curr. Enzyme Inhib.* 4 18–28. 10.2174/157340808783502540

[B24] Ghosh J. Myers C. E. (1997). Arachidonic acid stimulates prostate cancer cell growth: critical role of 5-lipoxygenase. *Biochem. Biophys. Res. Commun.* 235 418–423. 10.1006/bbrc.1997.6799 9199209

[B25] Guo F. Nimmanapalli R. Paranawithana S. Wittman S. Griffin D. Bali P. (2002). Ectopic overexpression of second mitochondria-derived activator of caspases (Smac/DIABLO) or cotreatment with N-terminus of Smac/DIABLO peptide potentiates epothilone B derivative–(BMS 247550) and Apo-2L/TRAIL–induced apoptosis. *Blood* 99 3419–3426. 10.1182/blood.V99.9.3419 11964312

[B26] Guo F. Wang Y. Liu J. Mok S. C. Xue F. Zhang W. (2015). CXCL12/CXCR4: a symbiotic bridge linking cancer cells and their stromal neighbors in oncogenic communication networks. *Oncogene* 35 816–826. 10.1038/onc.2015.139 25961926

[B27] Guo Q. Tian X. Yang A. Zhou Y. Wu D. Wang Z. (2014). Orientin in *Trollius chinensis* Bunge inhibits proliferation of HeLa human cervical carcinoma cells by induction of apoptosis. *Monatsh. Chem. Chem. Mon.* 145 229–233. 10.1007/s00706-013-1011-x

[B28] Houghton P. Fang R. Techatanawat I. Steventon G. Hylands P. J. Lee C. C. (2007). The sulphorhodamine (SRB) assay and other approaches to testing plant extracts and derived compounds for activities related to reputed anticancer activity. *Methods* 42 377–387. 10.1016/j.ymeth.2007.01.003 17560325

[B29] Isenberg J. S. Klaunig J. E. (2000). Role of the mitochondrial membrane permeability transition (MPT) in rotenone-induced apoptosis in liver cells. *Toxicol. Sci.* 53 340–351. 10.1093/toxsci/53.2.340 10696782

[B30] Jia L. Patwari Y. Kelsey S. M. Srinivasula S. M. Agrawal S. G. Alnemri E. S. (2003). Role of Smac in human leukaemic cell apoptosis and proliferation. *Oncogene* 22 1589–1599. 10.1038/sj.onc.1206322 12642862

[B31] Joseph B. Raj S. J. (2011). Pharmacognostic and phytochemical properties of *Ficus carica* Linn–an overview. *Int. J. PharmTech Res.* 3 8–12.

[B32] Kuete V. Kamga J. Sandjo L. P. Ngameni B. Poumale H. M. Ambassa P. (2011). Antimicrobial activities of the methanol extract, fractions and compounds from *Ficus polita* Vahl.(Moraceae). *BMC Complement. Altern. Med.* 11:6. 10.1186/1472-6882-11-6 21269424 PMC3037948

[B33] Kuo Y. H. Lin H. Y. (2004). Two novel triterpenes from the leaves of *Ficus microcarpa*. *Helv. Chim. Acta* 87 1071–1076. 10.1002/hlca.200490097

[B34] Lin J. P. Yang J. S. Lin J. J. Lai K. C. Lu H. F. Ma C. Y. (2012). Rutin inhibits human leukemia tumor growth in a murine xenograft model in vivo. *Environ. Toxicol.* 27 480–484. 10.1002/tox.20662 21254320

[B35] Liu M. Hai A. Huang A. T. (1993). Cancer epidemiology in the Far East–contrast with the United States. *Oncology* 6 99–110. 8318362

[B36] National Cancer Registry Ministry of Health Malaysia (2011). *Malaysia National Cancer Registry Report 2007.* Kuala Lumpur: National Cancer Registry, Ministry of Health Malaysia.

[B37] Oliveira A. P. Baptista P. Andrade P. B. Martins F. Pereira J. A. Silva B. M. (2012). Characterization of *Ficus carica* L. cultivars by DNA and secondary metabolite analysis: is genetic diversity reflected in the chemical composition? *Food Res. Int.* 49 710–719. 10.1016/j.foodres.2012.09.019

[B38] Rådmark O. Werz O. Steinhilber D. Samuelsson B. (2007). 5-Lipoxygenase: regulation of expression and enzyme activity. *Trends Biochem. Sci.* 32 332–341. 10.1016/j.tibs.2007.06.002 17576065

[B39] Ragasa C. Y. Tsai P. Shen C. C. (2009). Terpenoids and sterols from the endemic and endangered Philippine trees, *Ficus pseudopalma* and *Ficus ulmifolia*. *Philipp. J. Sci.* 138 205–209.

[B40] Ramamurthy S. Kumarappan C. Dharmalingam S. R. Sangeh J. K. (2014). Phytochemical, pharmacological and toxicological properties of *Ficus deltoidea*: a review of a recent research. *Annu. Res. Rev. Biol.* 4:2357 10.9734/ARRB/2014/8281

[B41] Ransohoff R. M. (2009). Chemokines and chemokine receptors: standing at the crossroads of immunobiology and neurobiology. *Immunity* 31 711–721. 10.1016/j.immuni.2009.09.010 19836265 PMC2787682

[B42] Ridley A. J. Schwartz M. A. Burridge K. Firtel R. A. Ginsberg M. H. Borisy G. (2003). Cell migration: integrating signals from front to back. *Science* 302 1704–1709. 10.1126/science.1092053 14657486

[B43] Santiago L. A. Mayor A. B. R. (2014). Lupeol: an antioxidant triterpene in *Ficus pseudopalma* Blanco (Moraceae). *Asian Pac. J. Trop. Biomed.* 4 109–118. 10.1016/S2221-1691(14)60218-5 25182281 PMC3819478

[B44] Shafi A. A. Yen A. E. Weigel N. L. (2013). Androgen receptors in hormone-dependent and castration-resistant prostate cancer. *Pharmacol. Therapeut.* 140 223–238. 10.1016/j.pharmthera.2013.07.003 23859952

[B45] Singh S. Singh U. P. Grizzle W. E. Lillard J. W. Jr. (2004). CXCL12–CXCR4 interactions modulate prostate cancer cell migration, metalloproteinase expression and invasion. *Lab. Invest.* 84 1666–1676. 10.1038/labinvest.3700181 15467730

[B46] Soga N. Connolly J. O. Chellaiah M. Kawamura J. Hruska K. A. (2009). Rac regulates vascular endothelial growth factor stimulated motility. *Cell Commun. Adh.* 8 1–13. 10.3109/15419060109080703 11775025

[B47] Somwong P. Suttisri R. Buakeaw A. (2013). New sesquiterpenes and phenolic compound from *Ficus foveolata*. *Fitoterapia* 85 1–7. 10.1016/j.fitote.2012.12.026 23274776

[B48] Srinivasula S. M. Hegde R. Saleh A. Datta P. Shiozaki E. Chai J. (2001). A conserved XIAP-interaction motif in caspase-9 and Smac/DIABLO regulates caspase activity and apoptosis. *Nature* 410 112–116. 10.1038/35065125 11242052

[B49] Tsai P.-W. De Castro-Cruz K. A. Shen C.-C. Chiou C.-T. Ragasa C. Y. (2012). Chemical constituents of *Ficus odorata*. *Pharm. Chem. J.* 46 225–227. 10.1007/s11094-012-0767-3

[B50] Uyub A. M. Nwachukwu I. N. Azlan A. A. Fariza S. S. (2010). In-vitro antibacterial activity and cytotoxicity of selected medicinal plant extracts from Penang Island Malaysia on metronidazole-resistant-*Helicobacter pylori* and some pathogenic bacteria. *Ethnobotany Res. Appl.* 8 095–106. 10.17348/era.8.0.95-106

[B51] Vanella L. Di Giacomo C. Acquaviva R. Barbagallo I. Cardile V. Kim D. H. (2013). Apoptotic markers in a prostate cancer cell line: effect of ellagic acid. *Oncol. Rep.* 30 2804–2810. 10.3892/or.2013.2757 24085108

[B52] Verhagen A. M. Ekert P. G. Pakusch M. Silke J. Connolly L. M. Reid G. E. (2000). Identification of DIABLO, a mammalian protein that promotes apoptosis by binding to and antagonizing IAP proteins. *Cell* 102 43–53. 10.1016/S0092-8674(00)00009-X 10929712

[B53] Vichai V. Kirtikara K. (2006). Sulforhodamine B colorimetric assay for cytotoxicity screening. *Nat. Protoc.* 1 1112–1116. 10.1038/nprot.2006.179 17406391

[B54] Vindrieux D. Escobar P. Lazennec G. (2009). Emerging roles of chemokines in prostate cancer. *Endocr. Relat. Cancer* 16 663–673. 10.1677/ERC-09-0109 19556286

[B55] Werz O. Steinhilber D. (2006). Therapeutic options for 5-lipoxygenase inhibitors. *Pharmacol. Therapeut.* 112 701–718. 10.1016/j.pharmthera.2006.05.009 16837050

[B56] Wynder E. L. Fujita Y. Harris R. E. Hirayama T. Hiyama T. (1991). Comparative epidemiology of cancer between the united states and Japan. A second look. *Cancer* 67 746–763. 1985768 10.1002/1097-0142(19910201)67:3<746::aid-cncr2820670336>3.0.co;2-1

[B57] Yang J. Liang Q. Wang M. Jeffries C. Smithson D. Tu Y. (2014). UPLC-MS-ELSD-PDA as a powerful dereplication tool to facilitate compound identification from small molecule natural product libraries. *J. Nat. Prod.* 77 902–909. 10.1021/np4009706 24617915 PMC4784093

[B58] Yang P. Cartwright C. A. Li J. Wen S. Prokhorova I. N. Shureiqi I. (2012). Arachidonic acid metabolism in human prostate cancer. *Int. J. Oncol.* 41 1495–1503. 10.3892/ijo.2012.1588 22895552 PMC3982713

[B59] Zakaria Z. A. Hussain M. K. Mohamad A. S. Abdullah F. C. Sulaiman M. R. (2012). Anti-Inflammatory activity of the aqueous extract of *Ficus deltoidea*. *Biol. Res. Nurs.* 14 90–97. 10.1177/1099800410395378 21278166

[B60] Zamzami N. Marchetti P. Castedo M. Zanin C. Vayssière J. L. Petit P. X. (1995). Reduction in mitochondrial potential constitutes an early irreversible step of programmed lymphocyte death in vivo. *J. Exp. Med.* 181 1661–1672. 10.1084/jem.181.5.1661 7722446 PMC2192017

[B61] Zheng S. Y. Li Y. Jiang D. Zhao J. Ge J. F. (2012). Anticancer effect and apoptosis induction by quercetin in the human lung cancer cell line A-549. *Mol. Med. Report* 5 822–826. 10.3892/mmr.2011.726 22200874

[B62] Zhou Y. Liu Y. E. Cao J. Zeng G. Shen C. Li Y. (2009). Vitexins, nature-derived lignan compounds, induce apoptosis and suppress tumor growth. *Clin. Cancer Res.* 15 5161–5169. 10.1158/1078-0432.CCR-09-0661 19671865 PMC2752044

