# Supplementary figures and images for: In Vitro Pro-apoptotic and Anti-migratory Effects of Ficus deltoidea L. Plant Extracts on the Human Prostate Cancer Cell Lines PC3
Source: Front Pharmacol. 2017 Dec 12;8:895. doi: 10.3389/fphar.2017.00895 (PMC5736988; doi:10.3389/fphar.2017.00895)

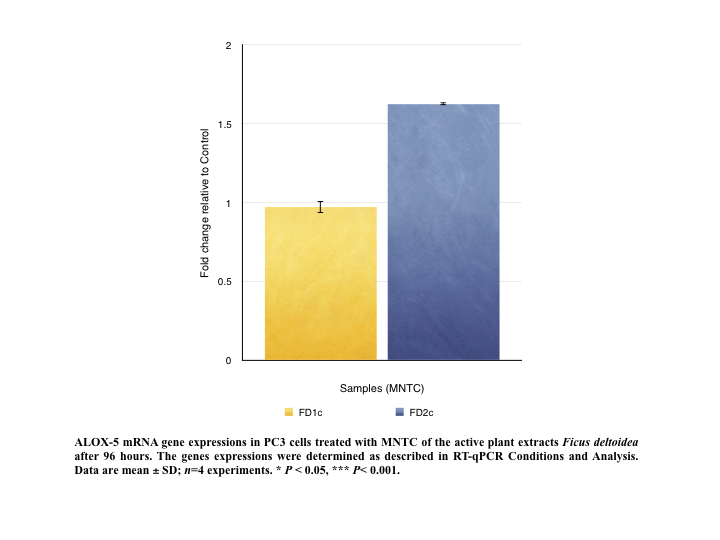

Supplement: Supplementary file 1 [file Image_1.TIF]

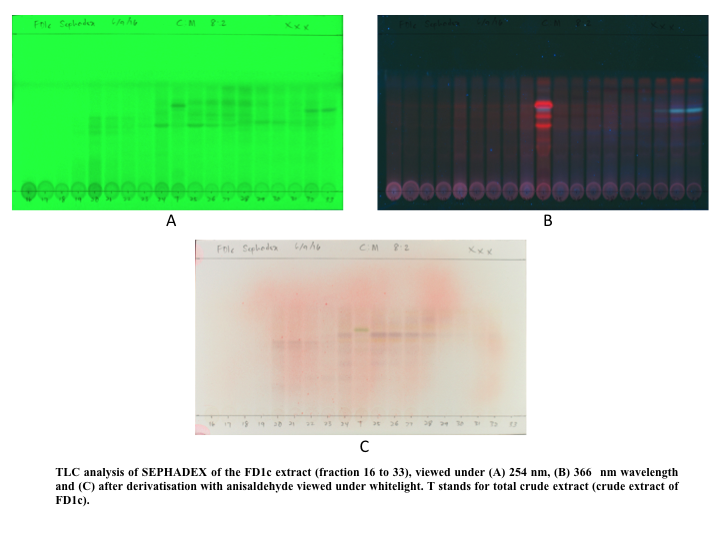

Supplement: Supplementary file 2 [file Image_2.TIF]

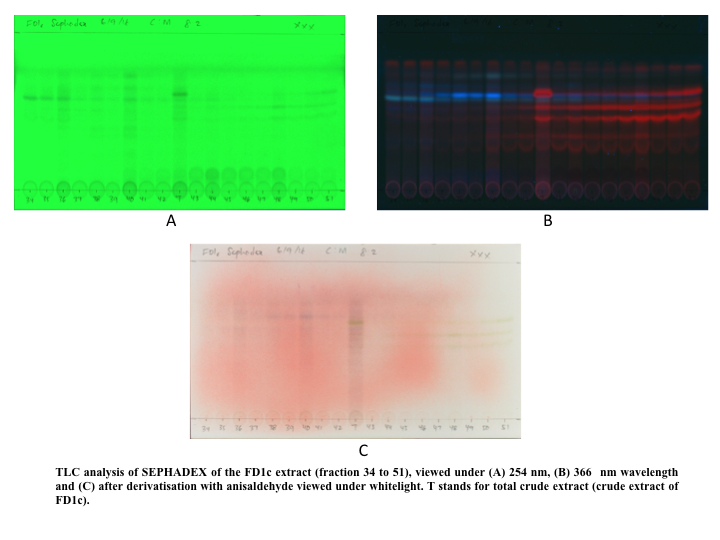

Supplement: Supplementary file 3 [file Image_3.TIF]

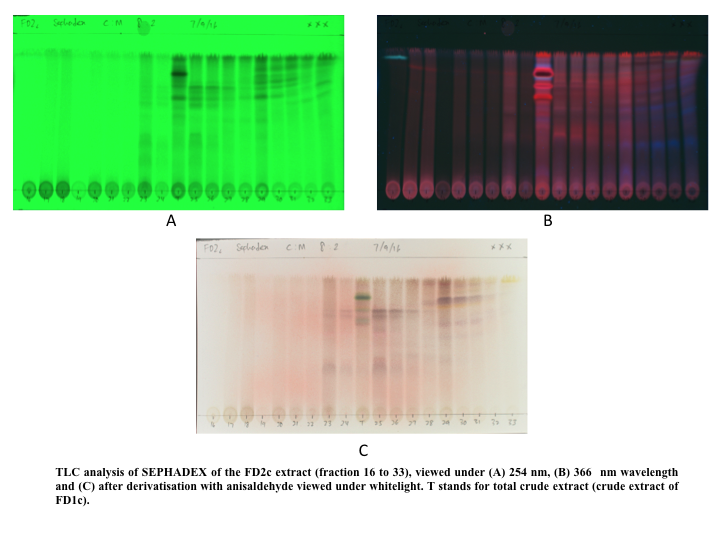

Supplement: Supplementary file 4 [file Image_4.TIF]

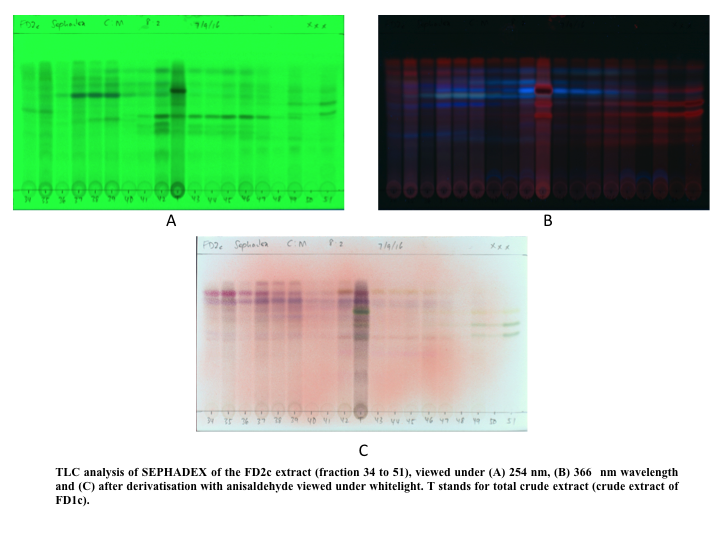

Supplement: Supplementary file 5 [file Image_5.TIF]

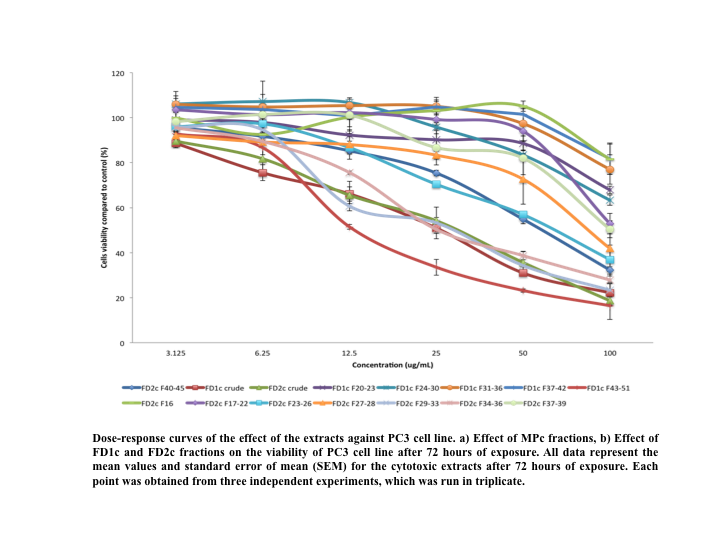

Supplement: Supplementary file 6 [file Image_6.TIF]

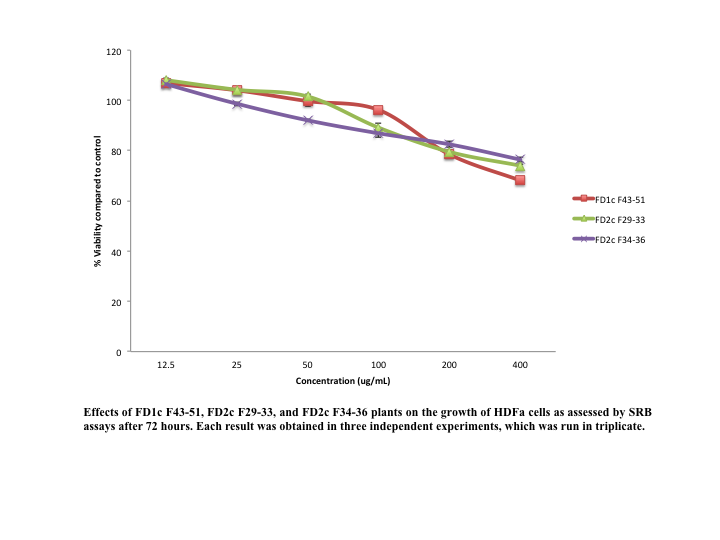

Supplement: Supplementary file 7 [file Image_7.TIF]
